# Effect of Phosphorylation on EGFR Dimer Stability Probed by Single-Molecule Dynamics and FRET/FLIM

**DOI:** 10.1016/j.bpj.2015.01.005

**Published:** 2015-03-10

**Authors:** Oana Coban, Laura C. Zanetti-Dominguez, Daniel R. Matthews, Daniel J. Rolfe, Gregory Weitsman, Paul R. Barber, Jody Barbeau, Viviane Devauges, Florian Kampmeier, Martyn Winn, Borivoj Vojnovic, Peter J. Parker, Keith A. Lidke, Diane S. Lidke, Simon M. Ameer-Beg, Marisa L. Martin-Fernandez, Tony Ng

**Affiliations:** 1 Richard Dimbleby Department of Cancer Research, King’s College London, London, UK; 2 Randall Division of Cellular and Molecular Biophysics, King’s College London, London, UK; 3 Science and Technology Facilities Council, Research Complex at Harwell, Rutherford Appleton Laboratory, Didcot, UK; 4 Gray Institute for Radiation Oncology & Biology, Department of Oncology, University of Oxford, Oxford, UK; 5 Division of Cancer Studies, King’s College London, London, UK; 6 Division of Imaging Sciences, King's College London, The Rayne Institute, St. Thomas Hospital, London, UK; 7 Computational Science and Engineering Department, Science and Technology Facilities Council, Rutherford Appleton Laboratory, Didcot, UK; 8 Cancer Research UK, London Research Institute, London, UK; 9 Department of Physics and Astronomy, University of New Mexico, Albuquerque, New Mexico; 10 Department of Pathology, University of New Mexico School of Medicine, Albuquerque, New Mexico; 11 Cancer Research and Treatment Center, University of New Mexico School of Medicine, Albuquerque, New Mexico

## Abstract

Deregulation of epidermal growth factor receptor (EGFR) signaling has been correlated with the development of a variety of human carcinomas. EGF-induced receptor dimerization and consequent trans- auto-phosphorylation are among the earliest events in signal transduction. Binding of EGF is thought to induce a conformational change that consequently unfolds an ectodomain loop required for dimerization indirectly. It may also induce important allosteric changes in the cytoplasmic domain. Despite extensive knowledge on the physiological activation of EGFR, the effect of targeted therapies on receptor conformation is not known and this particular aspect of receptor function, which can potentially be influenced by drug treatment, may in part explain the heterogeneous clinical response among cancer patients. Here, we used Förster resonance energy *transfer/*fluorescence lifetime imaging microscopy (FRET/FLIM) combined with two-color single-molecule tracking to study the effect of ATP-competitive small molecule tyrosine kinase inhibitors (TKIs) and phosphatase-based manipulation of EGFR phosphorylation on live cells. The distribution of dimer on-times was fitted to a monoexponential to extract dimer off-rates (*k*_off_). Our data show that pretreatment with gefitinib (active conformation binder) stabilizes the EGFR ligand-bound homodimer. Overexpression of EGFR-specific DEP-1 phosphatase was also found to have a stabilizing effect on the homodimer. No significant difference in the *k*_off_ of the dimer could be detected when an anti-EGFR antibody (425 Snap single-chain variable fragment) that allows for dimerization of ligand-bound receptors, but not phosphorylation, was used. These results suggest that both the conformation of the extracellular domain and phosphorylation status of the receptor are involved in modulating the stability of the dimer. The relative fractions of these two EGFR subpopulations (interacting versus free) were obtained by a fractional-intensity analysis of ensemble FRET/FLIM images. Our combined imaging approach showed that both the fraction and affinity (surrogate of conformation at a single-molecule level) increased after gefitinib pretreatment or DEP-1 phosphatase overexpression. Using an EGFR mutation (I706Q, V948R) that perturbs the ability of EGFR to dimerize intracellularly, we showed that a modest drug-induced increase in the fraction/stability of the EGFR homodimer may have a significant biological impact on the tumor cell’s proliferation potential.

## Introduction

The human epidermal growth factor receptor (EGFR, also known as HER or ErbB) family comprises four receptor tyrosine kinases (TKs) that play a key role in signaling in normal (1,2) as well as carcinogenic cells of various organs (3). Invasion and progression of some malignant tumors are frequently associated with overexpression of HER family members and amplification of downstream signaling pathways. In particular, EGFR (HER1 or ErbB1) overexpression has been linked to the progression of many human cancers, including head and neck, breast, and non-small-cell lung cancers (4,5), making it a primary candidate for targeted therapies.

EGFR is a 170 kDa transmembrane protein with intrinsic TK activity. Ligand binding to the extracellular region (domains I–IV) triggers conformational changes that expose domain II of EGFR, which unfolds into a dimerization arm (6). The newly accessible molecular interface allows for typical homo- or hetero-oligomeric interactions of the receptors across the entire family. This process is thought to drive the allosteric activation of the intracellular TK domain (TKD) and *trans* autophosphorylation of several cytoplasmic tyrosine residues within the dimer. These phosphorylated residues serve as docking sites for various adaptor molecules that are responsible for the propagation of downstream signaling (7).

A significant understanding of these conformation-dependent activation mechanisms has been facilitated by structure determination using x-ray crystallography (8–11) combined with site-directed mutagenesis (6,12–17) and pharmacological inhibition approaches (18–20). Crystallographic structures have shown an asymmetric, ligand-induced, active kinase dimer that forms through the C-lobe of one kinase molecule docking to the N-lobe of another kinase molecule (12,13). The juxtamembrane (JM) domain and C-tail segments, which flank the N- and C-terminal ends of the kinase domain, respectively, were also identified to regulate the activation process (12,13,21–23).

Single-molecule fluorescence imaging and spectroscopy methods, which can directly visualize the EGFR homodimer association/dissociation in real time (24–31), can provide invaluable insights into the static structures of various conformational states and their functional consequences. Experiments performed at the individual-molecule level allow us to observe the kinetic pathways of EGFR molecules in monomeric, dimeric, and different oligomeric states that are difficult to detect in ensemble measurements. Other investigators and we (32–34) have previously reported on the conformational changes induced by the anti-EGFR TK inhibitors (TKIs) erlotinib and gefitinib on EGFR. These recent findings suggest that TKI-bound receptors may exhibit different dissociation rates (*k*_off_) as a consequence of cytoplasmic phosphorylation downmodulation. Inside-out signaling is known to occur and may account for the stabilizing effect of type I inhibitors (which are thought to bind only the active EGFR-TKD conformation) such as erlotinib and gefitinib on the EGFR ectodomain (ECD) dimers (35). Various computational models have been developed to elucidate the full mechanism of the ErbB conformational changes that occur in response to growth factor stimulation or TKI treatment (36–43). Although there are some mathematical models of EGFR signaling that incorporate the single-molecule dynamics of EGFR interactions (44), advances are still limited by the scarcity of experimental data on the binding kinetics (*k*_on_ and *k*_off_) of intact receptors in live cells.

In this study, we used two-color, single-molecule imaging to follow the dissociation of EGFR dimers on the plasma membrane of epithelial breast cancer cells in real time and to characterize changes in the dissociation rate constant as a function of the receptor’s cytoplasmic phosphorylation state. Similar dissociation constants were determined for EGF and 425 Snap scFv ligand-bound dimers regardless of whether or not the ligand was causing EGFR activation. EGFR homodimers were stabilized by pretreatment with TKIs or in the presence of overexpressed density-enhanced phosphatase-1 (DEP-1). We recently showed that the transmembrane receptor protein phosphatase DEP-1 interacts with the intracellular portion of EGFR (45). Besides changes in single-molecule binding kinetics, ensemble Förster resonance energy transfer/fluorescence lifetime imaging microscopy (FRET/FLIM) measurements showed an increase in the fraction of receptor undergoing dimerization in cells that were pretreated with TKI. Interactions among individual monomers were also probed via single-pair FRET (spFRET). This study establishes the cytoplasmic tyrosine phosphorylation as a modulator of EGFR dimerization and oligomerization.

## Materials and Methods

### Cell culture and transfection

Cultured epithelial breast cancer cells (HCC1954) (46) were grown in RPMI 1640 (Sigma-Aldrich, UK) supplemented with 10% fetal bovine serum, 20 mM glutamine, and 1% penicillin/streptomycin (Invitrogen, Carlsbad, CA) under 5% CO_2_ at 37°C. The plasmid encoding GFP-tagged DEP-1 phosphatase (wild-type (WT) and phosphatase-incompetent DEP-1 cs mutant) was constructed and expressed as reported previously (45). Transient phosphatase expression in HCC1954 cells was achieved by transfection of 1 *μ*g of plasmid DNA using fugene HD (Promega, UK) according to the manufacturer’s protocol. The WT and I706Q V948R ErbB1 mutant plasmids were constructed and expressed as described previously (47). Transient transfection was achieved using the same protocol as for the phosphatase expression.

### Antibody fragments, ligands, and inhibitors

425(scFv) is derived from the mouse monoclonal antibody 425 directed against EGFR. The 425 Snap (scFv) expression vector was kindly provided by S. Barth (RWTH Aachen University, Aachen, Germany). Production and labeling of the 425 Snap (scFv) fusion protein (scFv in short) was performed as described elsewhere (48). The antibody was fluorescently labeled with either Snap-Alexa Fluor 546 or Snap-Alexa Fluor 647 (New England Biolabs, UK) with a labeling stoichiometry of 0.3 and 0.9 dye molecules/protein, respectively. Monoclonal mouse F4 antibody raised against the cytoplasmic domain of human EGFR was obtained from Cancer Research UK. The F4 antibody was amino labeled in-house with Alexa Fluor 546 (dye/protein = 1.1) and DyLight 649 (dye/protein = 3.1). Alexa Fluor 555 D38-B1 (anti-EGFR) and Alexa Fluor 647 D38-B1 (anti-EGFR) monoclonal antibodies raised against the intracellular part of the receptor were purchased from New England Biolabs, UK. EGF (Peprotech, NJ) was labeled (Cambridge Research Biochemicals, UK) with Alexa Fluor 546 and Atto 647 monoreactive esters (Life Technologies, UK) with dye/protein = ∼1. The TKIs lapatinib and gefitinib were purchased from VWR International (UK).

### Cell proliferation

Cells were plated at 0.5 × 10^4^ cells/well in a 96-well plate. The next day, they were transfected with 0.3 *μ*g WT and I706Q V948R ErbB1 plasmids per well. Gefitinib treatment was applied at 24 h after transfection at variable concentrations between 0 and 30 *μ*M. Cell viability was quantified at 96 h after gefitinib treatment using an Alamar Blue assay (Life Sciences). The Alamar Blue fluorescence readout was measured on a fluorescence plate reader and the dose-response curves of gefitinib treatment were determined.

### Sample preparation

#### Measurements in live cells

HCC1954 cells were seeded onto borosilicate glass-bottomed chambers at an appropriate number to reach ∼60–70% confluence within 24 h. After incubation in Optimem reduced serum media (Gibco, UK) for 2 h at 37°C under 5% CO_2_, the cells were treated with the desired drug (10 *μ*M gefitinib or lapatinib) for 1 h at 37°C. They were then incubated for 10 min at ambient temperature with two-color labeled EGF (∼300 pM Alexa Fluor 546 EGF and Alexa Fluor 647 EGF) before imaging was conducted. Biotinylated EGF-Streptavidin QD complexes at 500 nM final concentration were preformed by incubation in TBS for 30 min on ice. The complex was diluted with Optimem to a concentration of 1 nM and added to the live cells right before imaging. All measurements in live cells were carried out in HEPES (25 mM)-buffered Optimem at ambient temperature. Although a temperature of 37°C for measurements would have been more similar to physiological conditions, we performed the experiments at ambient temperature to slow down the ligand-induced EGFR internalization process, which would have rapidly depleted the membrane-bound receptor populations for our total internal reflection fluorescence (TIRF)-based single-molecule measurements. To enable quantitative single-molecule data analysis, room temperature was chosen as an adequate alternative to 37°C. However, all TKI pretreatments were performed at 37°C by incubation with 10 *μ*M inhibitor for 1 h under 5% CO_2_.

#### Measurements in fixed cells

Cells were cultured and seeded as described above on glass coverslips placed in 24-well plates. Fluorescence immunostaining was performed 24 h after plating. Cells were stimulated with 100 ng/ml (∼16 nM) EGF for 15 min at 37°C, chemically cross-linked with 4% paraformaldehyde for 30 min on ice, and permeabilized with 0.4% Triton X-100 for 10 min at room temperature. Samples were incubated overnight at 4°C with fluorescently labeled primary antibodies against EGFR (10 *μ*g/mL) and mounted in Mowiol or FluorSave (Calbiochem, UK) for FLIM and TIRF microscopy (TIRFM) imaging, respectively.

### FLIM

Time-domain FLIM data were acquired via a time-correlated single photon counting (TCSPC) custom-built, automated, open microscope (49). Briefly, this consisted of a white-light supercontinuum laser (SC450; Fianium, UK) for fluorescence excitation, a dual-axis scanner, a photomultiplier detector (PMH-100; Becker and Hickl, UK) and SPC electronics (SPC830; Becker and Hickl, UK). Images were acquired using an air 20× Plan Fluor objective (NA = 0.50; Nikon Instruments, UK). Fluorescence lifetimes were determined at every pixel using a modified Levenberg-Marquardt fitting technique as described previously (50). The FLIM images were batch analyzed by running an in-house exponential fitting algorithm (TRI2 software) written in LabWindows/CVI (National Instruments, Austin, TX). The fitting parameters for each time-resolved intensity image were recorded in individual output files and used to generate a distribution of lifetime and an average fluorescence lifetime. FLIM/FRET analysis was performed to investigate protein-protein interactions (51–54). Using the donor lifetimes only, FRET efficiencies were calculated based on the equation *E* = 1 − *τ*_*DA*_/*τ*_*D*_, where *τ*_*D*_ and *τ*_*DA*_ are the measured fluorescence lifetimes of the donor in the absence and presence of the acceptor, respectively.

### Single-molecule TIRFM

Single-molecule images were acquired using an objective-type TIRF setup based on a Nikon Ti Eclipse microscope (Nikon Instruments, UK) equipped with a 60×, NA = 1.49, Nikon TIRF objective. A continuous-wave, diode-pumped, solid-state laser with emissions at 527 nm (DTL313; Laser2000, UK) and a 642 nm continuous-wave laser diode (Stradus Vortran; Laser2000), respectively, were used as excitation sources and were coupled to the microscope with free-space optics and a computer-driven positioner (150-811ST; Thorlabs, UK) to control the TIRF angle. Fluorescence emission collected through the objective was split into two spectrally distinct channels by passing it through a dichroic mirror (Semrock FF 665-Di02; Semrock) and two band-pass filters (Bright Line 692/40 and 575/25; Semrock). The two emission channels were simultaneously recorded at a rate of 10 frames/s using an electron-multiplying charged-coupled device (EMCCD) camera (Evolve 512; Photometrics, UK). The individual microscope components were controlled using an in-house-written LabVIEW (National Instruments, Austin, TX) program.

Emission of GFP-tagged cells was excited at 470 nm via an LED laser diode (M470L2; Thorlabs), collected though the same objective, passed through the Nikon FITC filter block (EX465-495, DM505), and detected with a Nikon DS QiMc camera mounted on the back port of the microscope. NIS-Elements software (Nikon Instruments, UK) was used to capture and export the images in .tif format.

### Single-molecule data analysis

We performed multicolor single-molecule detection and tracking using previously described algorithms (52). Channel registration was extended to include a cubic polynomial transformation between the channels. We detected individual molecules using a single-channel feature detection algorithm by comparing two models for the region of interest (ROI) around each pixel. One model assumed that the image region around a pixel is described by pure noise, *H*_0_ (background parameter *B*_0_). Alternatively, when a molecule was present, the image region around a pixel was described by a feature profile from a single point emitter within the pixel plus background emission and stochastic noise, *H*_1_ (background parameter *B*_1_, feature intensity *I*, feature coordinates *x* and *y*). Given a specific description for each pixel of the background emission, stochastic noise, and feature profile, we calculated the probabilities for each model in each pixel using Bayes’ theorem, a process known as Bayesian segmentation. In this work, the feature profile given by the point spread function of the microscope was approximated by a Gaussian profile with a fixed, known width. The ROI considered around each pixel was a square with a side size of approximately four times the assumed feature profile’s full width at half-maximum intensity.

Fluorescence intensity time traces were generated using single proximity tracking. A detected feature, *i*, detected in the first frame seeded subsequent frames for which a connection probability that feature *i* belongs to track *j* was calculated. Features were assigned to tracks, with at most one feature per track and one track per feature based on the above calculated probabilities. Features that were not assigned to existing tracks seeded new tracks starting from the frame in which they were first detected. With the assumption that features are more likely to be linked to tracks in the closest spatial and temporal proximity, this method generates a set of tracks, each of which corresponds to a time series of the detected molecule parameters (intensity, *x* and *y* localization). Some tracks may exhibit missing frames due to blinking or nondetected features. A simple method allows one to follow the tracks through such gaps. We determined the position coordinates of each detected feature with subpixel resolution by fitting the image of each molecule to a two-dimensional Gaussian function.

We analyzed the extracted tracks for temporal coincidence using custom software in LabVIEW. A homodimer was identified when the feature detected in one channel was persistently located within 1 pixel (160 nm) from a feature in the second channel. Single fluorophores were typically detected with signal/noise ratio (SNR) ≅ 8, for which we estimated an average localization uncertainty of ∼80 nm (Fig. S1 in the Supporting Material). The separation distance between the two monomers detected in the green and red channels, respectively (calculated as the Euclidian distance between the (*x*, *y*) coordinates of the feature detected in the green and red channels at the same time point *t*) was plotted as a function of time. To improve the SNR in separation distance between molecules and, by implication, more accurately determine the duration of a dimerization event, we applied a five-point moving-average smoothing filter (10 frames/s). Additionally, tracks with more than four consecutive frames in which a molecule was not detected were not included in the analysis, since the motion of the molecules in such gaps is unknown. We found that gaps of fewer than four time points allowed for fluorophore blinking and for the same molecule to be picked up as it reappeared at the same location. The event duration was measured in a similar manner as previously described (55). Dimer dissociation was marked by a transition in separation distance beyond the threshold value of 160 nm (1 pixel). The average duration of the dimerization event, *τ*_on_, was determined by fitting an exponential decay to the histogram of dimer association times using a Marquardt minimization algorithm. The monoexponential function was defined as *f* (*t*) = *Z* + *A*e^−*t*/*τ*^_on_, where *Z* is a baseline offset and *A* is the amplitude of the exponential function. Fitting was performed with the baseline offset constraint to be positive. The error of the decay, Δ*τ*_on_, was determined from the Levenberg-Marquardt covariance matrix as previously described (56). The dissociation rate *k*_off_ was determined as 1/*τ*_on_ with the error Δ*k*_off_ = Δ*τ*_on_/*τ*^2^_on_. Correlated motion analysis between pairwise single-molecule trajectories was performed as previously described (24,57,58). The initial separation distance between two-color EGFR trajectories was determined and if it fell below the specified cutoff, the pair was further analyzed in sequential frames. To better describe the receptor motion, we calculated two parameters: the magnitude of a receptor’s displacement (called the jump magnitude) and the extent of correlated motion between nearby receptors (called the uncorrelated jump distance). If two receptors formed a transient dimer, their diffusion would be significantly reduced, which would show in a decrease in their jump magnitude. Additionally, if two receptors formed a transient dimer, they would move together, i.e., the motion of the two interacting receptors would be correlated, producing a decrease in the uncorrelated jump distance. By plotting the jump magnitude and the uncorrelated jump distance as a function of distance between receptor pairs, we can detect the presence of transient dimers, provided their lifetime is longer than the imaging acquisition time.

To determine spFRET molecules, we used the multichannel feature detection as described previously (27,59). In this case, a two-channel feature was identified when a molecule was present at a particular location in the green and/or red channels. Additional software filters were introduced to ensure that the donor and acceptor time traces were at least 50% anticorrelated, no neighboring molecules were located within 1 pixel, and the displacement during the measurement was less than 0.8 pixels.

## Results

### Enhanced EGFR homodimerization induced by blocking tyrosine phosphorylation

**Figure 1:**
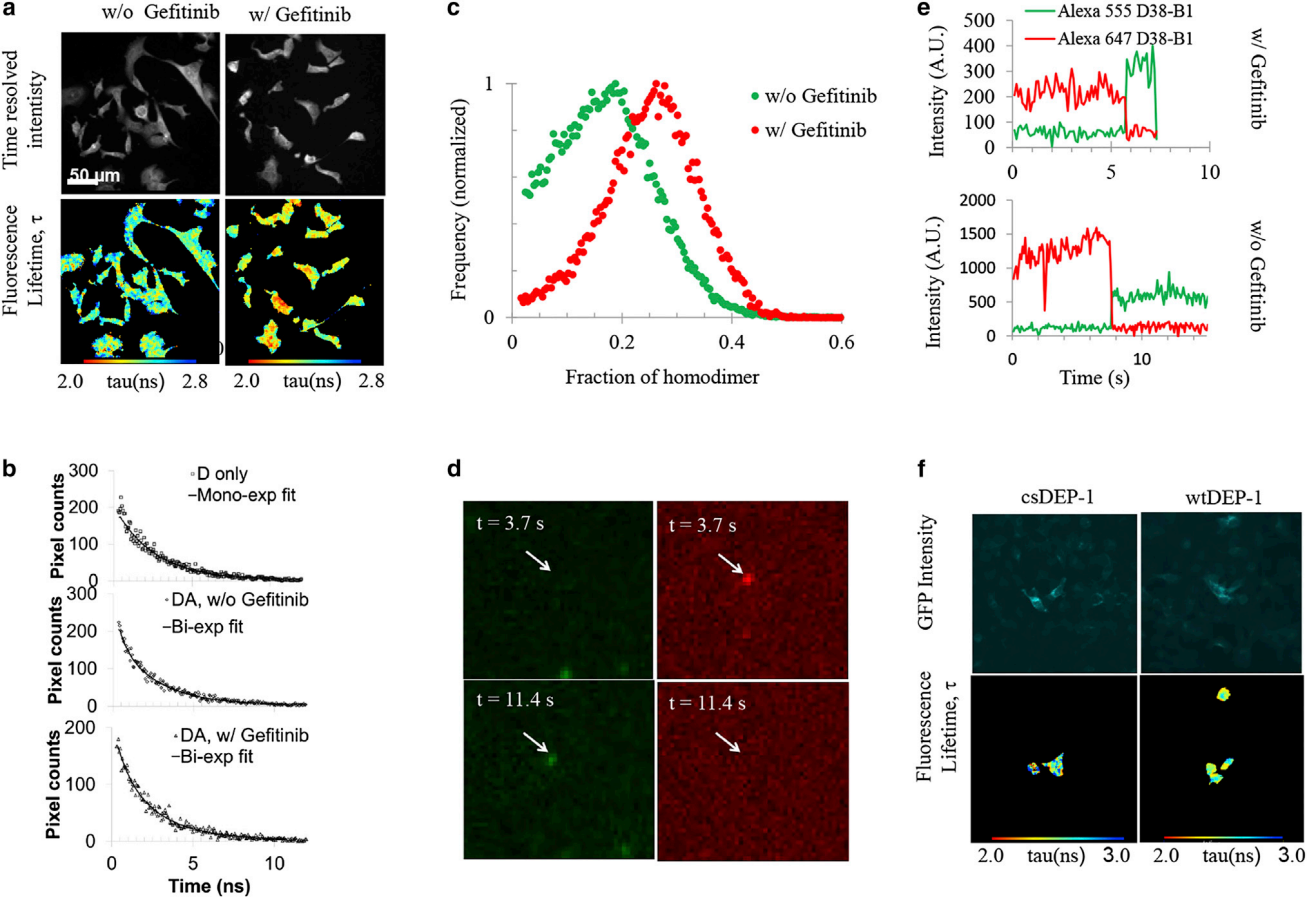
Effect of gefitinib on EGFR homodimerization. (*a*) FRET/FLIM images of HCC1954 cells that were stimulated with EGF in the absence or presence of gefitinib pretreatment, and then fixed and stained with Alexa 546- and Dy Light 649-labeled antibodies (clone F4) that recognize the intracellular domain of EGFR. This corresponds to an increase in the apparent average FRET efficiency from 1.2% without gefitinib treatment (*τ*^EGF^ = 2.46 ± 0. 19 ns) to 7.5% with gefitinib treatment (*τ*^gefitinib+EGF^ = 2.30 ± 0.17 ns; *p* < 0.001). Fluorescence lifetimes were calculated by a monoexponential fit to the data. Scale bar is 50 *μ*m for all images. (*b*) Mono- and biexponential pixel fittings of the individual FRET/FLIM images of D-only-labeled cells (Alexa 546) and DA (Alexa 546, Dy Light 649)-labeled cells before and after gefitinib treatment, respectively. (*c*) The partial contributions of the noninteracting, D-only-labeled species and interacting DA-labeled species were deconvoluted and histograms of the EGFR homodimers subpopulations before (*green*) and after (*red*) gefitinib treatment were plotted. Fractional-intensity histograms were accumulated over five images and normalized for direct comparison. The homodimer percentage increased from 17% to 24% after the gefitinib treatment, which corresponds to ∼40% enhanced dimerization. (*d*) Single-molecule signature FRET images of an individual molecule. Single-molecule images of EGF-stimulated fixed cells fluorescently labeled with the Alexa Fluor 555/Alexa Fluor 647 D38-B1 FRET pair were acquired with dual-excitation TIRF (Fig. S2 b). At *t* = 3.7 s, only the acceptor emission can be detected. At *t* = 11.4 s, the donor signal arises upon acceptor photobleaching. Scale bar is 1 *μ*m for all images. (*e*) Corresponding spFRET track showing anticorrelated D and A signals (E = 98%). A similar track was recorded for a gefitinib-pretreated molecule (E = 81%). (*f*) GFP fluorescence and donor FLIM images (of Alexa 546-labeled anti-EGFR antibody) of fixed HCC1954 cells transfected with GFP cs DEP-1 (Alexa 546 *τ*^csDEP-1^ = 2.69 ± 0. 23 ns) and WT DEP-1 (Alexa 546 *τ*^WTDEP-1^ = 2.45 ± 0. 11 ns) plasmids for inactive and overexpressed DEP-1 (EGFR) phosphatase, respectively, without EGF stimulation. The apparent average FRET efficiency increased from 3% for inactive DEP-1 phosphatase to 5% for overexpressed DEP-1 (*p* < 0.001). To see this figure in color, go online.

To investigate the regulatory role of cytoplasmic autophosphorylation in EGFR signaling, we measured the average fluorescence lifetime of EGF-bound receptor with and without TKI pretreatment. EGFR was labeled with cytoplasmic Alexa Fluor 546 F-4 as donor (D) and DyLight 649 F-4 as acceptor (A). Fig. 1 *a* shows the intensity and lifetime images of EGF-stimulated cells with and without gefitinib pretreatment. Histograms of donor lifetimes in the presence of acceptor for EGF-bound receptors with and without gefitinib pretreatment were fitted to a monoexponential, yielding *τ*_Gefitinib +EGF_^D,A^ = 2.30 ± 0.17 ns and *τ*_EGF_^D,A^ = 2.46 ± 0.19 ns, respectively. The errors are the standard deviations determined for an individual measurement averaged over five measurements. All of the experimentally determined parameters reported here were statistically tested by paired *t*-test with a significance level of 0.5. Gefitinib pretreatment was found to be associated with a statistically significant reduction in lifetime (*p* < 0.001). Lapatinib-pretreated cells exhibited a fluorescence lifetime of *τ*_Lapatinib +EGF_^D,A^ = 2.47 ± 0.15 ns, which was not statistically significant from that of untreated control cells (data not shown). We note that gefitinib is known to bind to the active kinase conformation of EGFR, whereas lapatinib binds to the inactive kinase conformation of EGFR and Her2. The different effects of gefitinib and lapatinib pretreatment are consistent with our previous findings in a different cell model (34). Control experiments on donor-only EGF-stimulated cells resulted in a fluorescence lifetime *τ*_EGF_^D^ = 2.49 ± 0.14 ns.

The measured donor lifetimes were averaged over a heterogeneous mixture of species. We assumed that the donor lifetime can be expressed as the sum of two subpopulations of molecules (*i* = 1, 2), where each of the subpopulations is characterized by a specific lifetime, *τ*_*i,*_, and amplitude, *A*_*i*_. The two subpopulations were represented by the noninteracting (monomer) and interacting (dimer) species, respectively. Fluorescence lifetimes were determined by pixel fitting or multi-image global fitting of the FRET/FLIM data as previously described (60). Results of this analysis are shown in Fig. 1 *b*. Lifetime *τ*_1_ was determined by monoexponential multi-image global fitting of the D-only-labeled cells to be 2.543 ns. The clear shift to lower lifetimes in the presence of gefitinib indicates the occurrence of FRET and hence increased dimer formation. The dimer lifetime, *τ*_2_, was calculated by biexponential multi-image global fitting of the DA/gefitinib-treated cells, with *τ*_1_ fixed to the previously determined value of 2.543, and *A*_1_ and *A*_2_ allowed to vary freely. We calculated the fluorescence lifetime of the gefitinib-treated homodimer as *τ*_2_ = 0.604 ns. After determining the lifetimes of the noninteracting and interacting species, we calculated their respective amplitudes *A*_*i*_ in both gefitinib-treated and untreated samples by biexponential pixel fitting of the data with fixed parameters *τ*_1_ = 2.543 ns and *τ*_2_ = 0.604 ns. Fitting was repeated for each image in a set of five. The fraction or subpopulation of dimers was determined as *A*_2_/(*A*_1_ + *A*_2_), where *A*_1_ and *A*_2_ are the previously determined fractions of monomer and dimer, respectively. The subpopulation of EGFR homodimers increased from 17% (0.174 ± 0.006) to 24% (0.244 ± 0.029) with gefitinib treatment. The errors are the standard deviation of the fractional intensity calculated over the set of five images. The relative increase in homodimerization with gefitinib pretreatment was found to be statistically significant (*p* < 0.01). Equivalently, gefitinib induced an ∼41% shift in the contribution of interacting species from a monomeric state toward a dimeric state, as shown in Fig. 1 *c*.

The above interpretation is further supported by our observations at the single-molecule level. Ensemble FRET measurements cannot distinguish whether the detected signal arises from dimers or higher-order oligomers, or detect heterogeneity in the distribution of FRET efficiencies. When using a dye/protein stoichiometry of one, one can use single-molecule methods to count the number of monomers (61) using one color only, or spFRET (62), which can provide detailed structural information about the molecules of interest. EGFR was tagged with the cytoplasmic antibody D38-B1 fluorescently labeled stochastically with Alexa Fluor 555 (D) and Alexa Fluor 647 (A). A detailed view of a single molecule experiencing spFRET is shown in Fig. 1 *d*. The corresponding fluorescence traces are plotted in Fig. 1 *e*. As can be seen in Fig. 1, *d* and *e*, at earlier times (3.7 s after the beginning of the measurement), a bright fluorescent molecule can be observed in the acceptor (red) channel, whereas there is no detectable signal at the same location in the donor (green) channel. At later times (11.4 s after the beginning of the measurement) and after acceptor photobleaching, the acceptor can no longer be detected in the red channel and the donor intensity increases in the green channel. Changes in spFRET for a gefitinib-pretreated molecule are also shown in Fig. 1 *d*.

Our data analysis revealed fluorescence traces displaying both multiple and single-step donor and/or acceptor photobleaching events. Since the antibodies used for immunofluorescence were stochastically labeled with dye per protein ratios between 2 and 4, we cannot attribute the multiple photobleaching steps traces to genuinely formed oligomers. However, single-step photobleaching traces demonstrate the EGFR-EGFR interaction. For the particular examples shown here (Fig. 1 *d*), we calculated spFRET efficiencies upon acceptor photobleaching according to the equation *E* = 1 – *I*_DA_/*I*_D_, where *I*_D_ and *I*_DA_ are the average donor fluorescence intensities in the absence and presence of acceptor, respectively. In contrast to calculations of FRET efficiency using acceptor emission, spFRET efficiencies calculated only from the donor emission do not need to be corrected for leakage of the Alexa Fluor 555 signal into the Alexa Fluor 647 channel, for direct excitation of Alexa Fluor 647, or for different quantum yields and quantum efficiencies of the two fluorophores. The spFRET efficiencies for the individual traces were calculated to be 98% (Fig. 1 *d*) and 81% (Fig. 1 *e*) for EGFR homodimers without and with gefitinib pretreatment, respectively. These correspond to D-A separation distances of ∼25 Å and 40 Å, respectively, assuming randomly oriented fluorophores and a 51 Å Förster radius for the FRET pair (Alexa Fluor 555, Alexa Fluor 647 fluorescently labeled anticytoplasmic EGFR antibodies). The measured distances are given as direct evidence of homodimer formation. The lack of site-specific dye labeling of the antibody prevented us from determining the specific separation distances between the EGFR monomers in these experiments. Assuming that the fluorescently labeled cytoplasmic antibodies bind to the two monomers in a TKD asymmetric dimer after TKI treatment (35), we can only infer (from the short D-A separation measured) that the antibodies’ binding sites (epitopes) are located close to the dimerization interface. Distance measurements based on the crystal structure of the asymmetric active dimer conformation of the TKD as reported by Protein Data Bank ID: 2JIU (20) have shown that the distance between the two epitopes can be between 10 Å and >100 Å depending on their exact location.

The high spFRET efficiencies calculated for the examples shown, together with the limited number of such observations we were able to make and the lower FRET efficiencies measured in the ensemble, support the idea of a heterogeneous population comprised of a small subpopulation of high-FRET-efficiency dimers and a majority of molecules noninteracting on the FRET range scale. These structurally different EGFR-EGF complexes may also account for the different ligand binding affinities previously reported (63,64).

Taking advantage of our previous finding that the EGFR-interacting protein phosphatase DEP-1 decreases EGFR phosphorylation and inhibits EGFR internalization and downstream signaling, we investigated the effect of its overexpression on the EGFR homodimerization level (45). Successfully transfected cells were manually selected for the analysis (Fig. 1 *f*). Surprisingly, similar values of the fluorescence lifetime were measured for EGF-stimulated, immobilized HCC1954 cells transfected with the GFP cs DEP-1 (*τ*_*EGF*_^csDEP-1^ = 2.55 ± 0. 20 ns) and WT DEP-1 (*τ*_*EGF*_^csDEP-1^ = 2.54 ± 0. 22 ns), respectively, and stained with the FRET pair (Alexa 546 and Cy5)-labeled intracellular antibody Ab15 (EGFR). In contrast to TKI pretreatment, when tyrosine phosphorylation was blocked before EGF stimulation, overexpression of DEP-1 phosphatase competed against the EGF stimulation and the two processes could cancel out in the ensemble. However, performing the assay in nonstimulated cells induced a significant (*p* < 0.001) drop in lifetime between the cells expressing the inactive cs DEP-1 (*τ*^csDEP-1^ = 2.69 ± 0. 23 ns) and overexpressing WT DEP-1 (*τ*^WTDEP-1^ = 2.45 ± 0. 11 ns), as shown in Fig. 1 *f*. To calculate the fraction of interacting species (homodimers), we performed a fractional-intensities analysis as previously described (60). Multi-image global monoexponential fitting of the Alexa 546 donor-only-stained cells transfected with the GFP cs DEP-1 plasmid yielded a fluorescence lifetime *τ*_1_ = 2.74 ns. The fluorescence lifetime for the interacting species, *τ*_2_, was derived by multi-image global fitting of the two-color (Alexa 546, Cy5) WT GFP-DEP1 transfected cells FRET/FLIM data to a biexponential with *τ*_1_ fixed to 2.74 ns. The fluorescence lifetime of the interacting species was determined to be 0.74 ns. The fractional intensity of the homodimer in the cs DEP-1 and WT DEP-1 transfected cells was derived from the biexponential pixel fits of all five respective images, with *τ*_1_ and *τ*_2_ fixed to the previously determined values. The fraction of homodimers in the cs DEP-1 transfected cells was determined to be 16%. Overexpression of DEP-1 phosphatase led to an increase in the fraction of homodimers to 20% in the WT DEP-1 transfected cells. Like gefitinib pretreatment, phosphatase overexpression facilitated dimer formation (with a 23% increase in the fraction of dimers), reinforcing the positive correlation observed between receptor dimerization and a dephosphorylated state.

### Two-color detection of EGFR dissociation and off-rate measurement in the plasma membrane

One possible explanation for the macroscopically measured increase in EGFR dimer formation in response to gefitinib treatment is the prolonged duration of homodimerization events in live breast cancer cells after pretreatment with gefitinib. Single-molecule methods have the ability to capture the dimerization process in real time and allow us to derive the receptor reaction kinetics and effect of gefitinib on the dissociation rate of the homodimer.

Endogenous association/dissociation events of EGFR receptors were observed in real time (10 frames/s) on the membrane of live HCC1954 cells that were provided with a mixture of EGF fluorescently labeled with two spectrally different fluorophores and allowed to bind their ligand. In a previous study, it was reported that fluorescent labeling of the N-terminal amino group of EGF did not to interfere with individual receptor dynamics (27). Here, single fluorophores were typically detected with an SNR ≅ 8, for which we estimated an average localization uncertainty of ∼80 nm (Fig. S1 f). Individual Alexa Fluor 546 EGF- and Atto 647 EGF-bound receptors diffusing on the plasma membrane were detected in the green and red channels, as illustrated in Fig. S2 a by the *green* and *red spots*, respectively. The green and red molecules located within one pixel (160 nm) of each other were considered to be colocalized in space and selected for *k*_off_ derivation.

**Figure 2:**
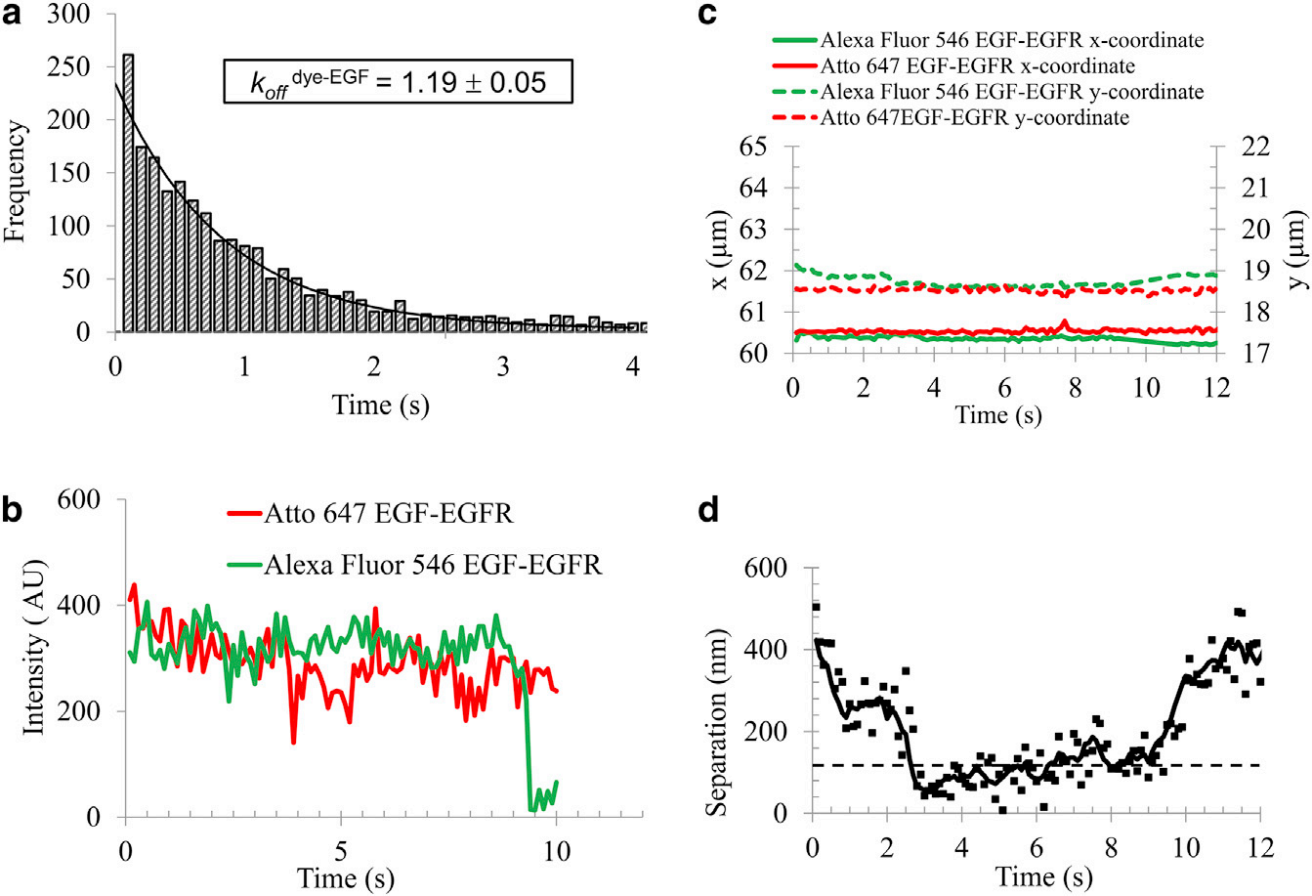
Kinetic analysis of EGFR homodimer dissociation. (*a*) Distribution of EGFR homodimer lifetimes after EGF stimulation for all accumulated data acquired in five individual experiments employing various TKIs (gefitinib, lapatinib, and AZD 8931). The distribution of more than 2000 dimerization events was fitted to a monoexponential where *k*_*app*_^EGF^ = 1.19 ± 0.05 s^−1^. (*b*) Two-color fluorescence emission corresponding to a pair of colocalized molecules labeled with Alexa Fluor 546 (*green*) and Atto 647 (*red*), respectively. (*c*) Changes with time of the horizontal and vertical positions of the dimerization partners. The two molecules temporally coexist between ∼0 and 12 s from the beginning of the measurement. Dimer formation implies simultaneous detection of both molecules at the same time (temporal colocalization) and same location (spatial colocalization). The horizontal and vertical feature positions report on the molecules’ spatial colocalization. (*d*) The separation distance between the two-color monomers in proximity to each other decreases below the threshold and marks the start of the dimerization event. For the duration of the dimerization event, the separation distance remains under the threshold, followed by a stepwise increase above the threshold upon dimer dissociation. This allows us to extract the apparent duration of an individual dimerization event, *τ*_app_. The photobleaching corrected colocalization duration provides the *τ*_on_ for the EGFR homodimer and the corresponding dissociation rate *k*_off_. To see this figure in color, go online.

The EGFR homodimer off-rates were determined from repeated individual experiments for specific conditions (Table S2). The histogram of dimer lifetimes shown in Fig. 2 *a* summarizes the two-color data for EGF-bound receptors acquired in a total of five individual experiments on more than 2000 dimerization events. A dissociation constant *k*_off_^EGF^ = 1.19 ± 0.05 s^−1^ was determined by fitting the distribution of lifetimes to a single exponential (55).

The *k*_off_ derivation was performed as follows: First, fluorescence intensities were used to detect two molecules. One molecule was labeled with Alexa Fluor 546 (green) and its dimerization partner was tagged with Atto 647 (red). Fig. 2 *b* shows full-time fluorescence traces of a pair of molecules that colocalized during the acquisition time. The duration of fluorescence emission for the full track length was compared with the duration of fluorescence for the length of the colocalized tracks. Full tracks that started or ended with simultaneous emission in the green and red channels or colocalized for the entire acquisition time were excluded from the analysis because the start and/or end of the dimerization event could not be clearly determined.

**Figure 3:**
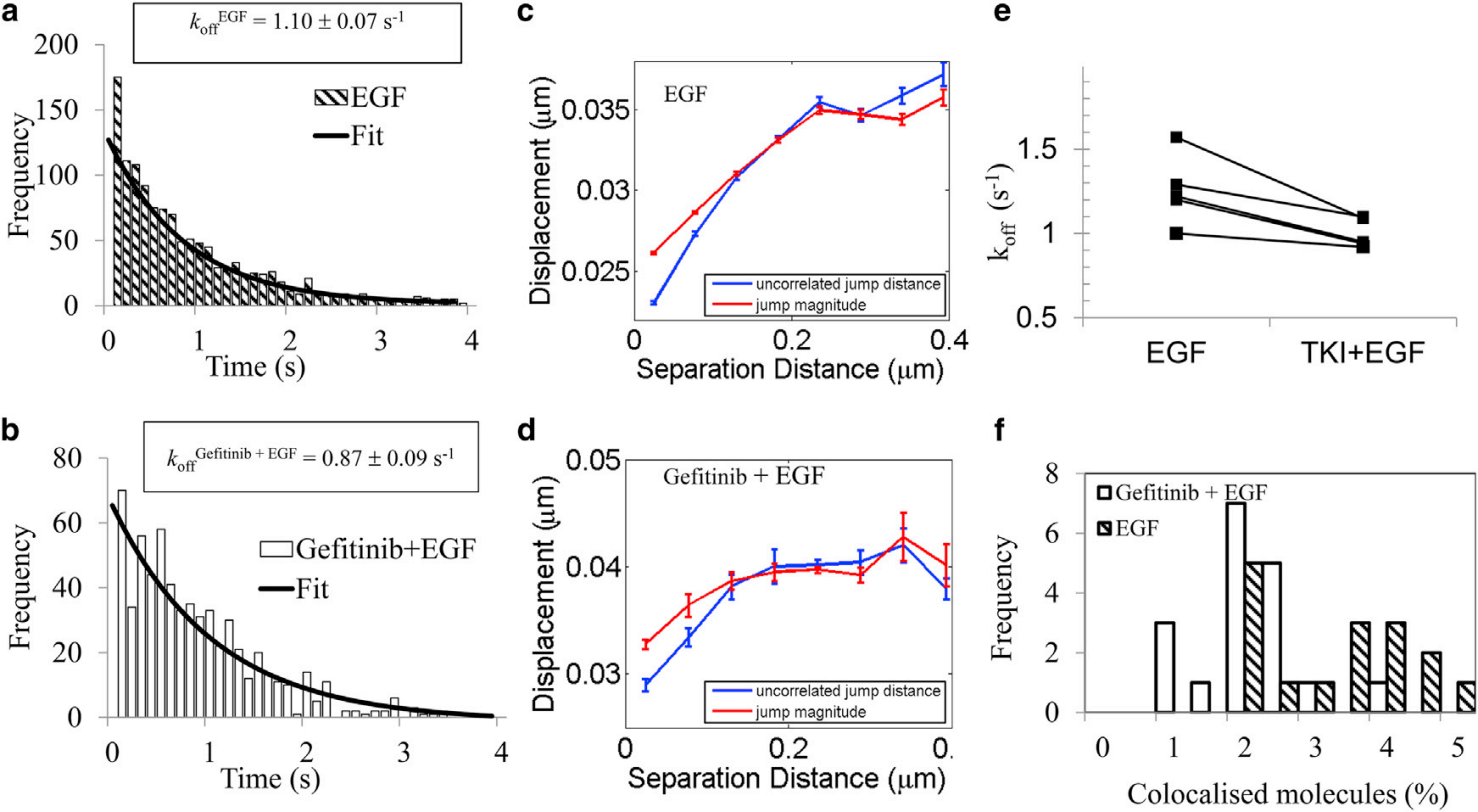
Single-molecule analysis of the effect of the TKI gefitinib on the EGFR homodimer stability. (*a* and *b*) Histograms of the duration of dimerization events fitted to a single exponential with *k*_off_^EGF^ = 1.10 ± 0.07 s^−1^ and *K*_off_^gefit+EGF^ = 0.87 ± 0.09 s^−1^ when cells were pretreated with gefitinib. (*c* and *d*) Ensemble correlated motion analysis of two-color single-particle tracking data for EGF-stimulated cells in the absence and presence of gefitinib pretreatment, respectively, for all two-color tracked molecules. The average receptor displacement (*red*) and the degree of uncorrelated motion (*blue*) are plotted as function of the separation distance. Correlated motion at small separation distances indicates dimerization. Increased correlation at short distances can be seen by the decrease in the uncorrelated jump distance (*blue*) when the two receptors approach one another. The diffusion behavior was monitored via the jump magnitude (*red*). Decreased mobility with dimerization (i.e., a drop in jump magnitude at short distances) was observed independently of gefitinib treatment. (*e*) Dissociation rates calculated from five individual experiments show a consistent reduction in *k*_off_ (*p* < 0.05) with TKI pretreatment independently of the type of TKI used (lapatinib, gefitinib, or AG 1478). (*f*) Decreased sample heterogeneity after gefitinib pretreatment. The fraction of colocalized molecules shifted from 3.59% ± 1.56% to 1.95% ± 0.63% after gefitinib pretreatment. To see this figure in color, go online.

The molecules were tracked for the duration of the measurement and their *x* and *y* coordinates were recorded. If the duration of the encounter was longer than 500 ms (five frames, i.e., the time during which the two molecules were within one pixel (160 nm) of each other), a dimerization event was deemed to have occurred. The duration of a single dimerization event or the lifetime of an individual dimer, *τ*_on_, was determined using the spatial and temporal information collected for a pair of colocalized molecules. Dimer events with durations between 0.5 s and 6 s were detected and included in the fit of the histogram. Fig. 2 *c* shows the variation in *x* and *y* coordinates for the two molecules during the time they were detected in the two channels (between 0 and 12 s from the beginning of the acquisition). Changes in the separation distance between the two molecules as a function of time are plotted in Fig. 2 *d*. As one of the molecules diffused away, the EGFR dimer dissociated and the separation distance increased abruptly above the threshold (Fig. 2 *d*). The 160 nm cutoff was empirically found to be the most robust given the SNR for our data. A variable dimerization cutoff value based on the localization uncertainty of individual pairs of colocalized molecules was also tested. However, this criterion was found to be less robust in the case of noisier data (larger localization uncertainties) when unwanted events were included in the analysis. The large cutoff value is comparable to the size of lipid raft nanodomains where receptors may be coconfined, but the existence of homodimeric rafts is very transient with lifetimes below our temporal resolution of 10 frames/s. The probability of tracking incidental colocalization or codiffusion for multiple frames is small. Our observations revealed spatial and temporal colocalization that was longer than the incidental colocalization but shorter than the time to photobleaching, indicating a higher likelihood that we mostly observed bona fide dimers (65). We also note that the average track lengths we measured were more than 10 times longer than the association times, which eliminates any concerns regarding molecule disappearance due to photobleaching on the timescale of the dimerization events. The photobleaching lifetimes for the fluorescent probes used were determined from the track lengths of the individual fluorophores in single-molecule colocalization experiments. The experimentally determined times to photobleaching were consistent with previously estimated values for single-molecule experiments (66). The decay constants were corrected for photobleaching as described elsewhere (67), providing the true colocalization duration and rates (Table S1). To further confirm the presence of EGFR dimers, we performed additional data analysis to determine the degree of correlated motion, as described briefly in Materials and Methods and elsewhere (24,58). We observed that the magnitude of the uncorrelated jump distance for receptor pairs decreased as the receptors were in close proximity (Fig. 3 *c*, *blue line*), consistent with the formation of dimers. The jump size also decreased at small separation distances (Fig. 3 *c*, *red line*), but not to the same extent as the uncorrelated jump distance, further supporting the notion that the effect is due to dimerization and not merely coconfinement. In this study, we preferred to use small organic fluorophores and take advantage of their photophysical characteristics, which allowed us to perform ensemble FRET/FLIM measurements on the endogenous receptor.

### ATP-competitive, small-molecule TKIs stabilize the EGFR dimer

We further used our single-molecule assay to assess the effect of various small-molecule TKIs (i.e., gefitinib, AG1478, and lapatinib) on EGFR homodimerization. TKIs compete with ATP for binding to the EGFR intracellular TK domain. Untreated and TKI-pretreated cells were incubated with picomolar concentrations of two-color EGF and imaged by TIRFM. Data were acquired from 20 different areas per experiment, with two to six cells in the field of view. The monoexponential fits of the EGFR homodimer lifetimes determined from an individual experiment are displayed in Fig. 3, *a* (nontreated cells) and *b* (gefitinib-treated cells). The measured EGFR dimer off-rates were *k*_off_^EGF alone^ = 1.10 ± 0.07 s^−1^ (1294 dimerization events) and *k*_off_^gefit+EGF^ = 0.87 ± 0.09 s^−1^ (634 dimerization events) in the absence and presence of gefitinib treatment, respectively.

Dimerization was again confirmed by correlated motion analysis (Fig. 3 *d*). The observed decrease in the uncorrelated jump distance at small separations demonstrates the presence of dimers in both untreated (Fig. 3 *c*) and gefitinib-treated (Fig. 3 *d*) cells.

The off-rates derived from five individual paired experiments are shown in Fig. 3 *e*. Consistent trends were observed in repeat experiments, regardless of which TKI was used. The dissociation rates, averaged over these five experiments (lapatinib and gefitinib were each used in two independent experiments and AG1478 was used in one experiment), decreased from *k*_off_^EGF alone^ = 1.22 ± 0.20 s^−1^ to *k*_off_^TKI+EGF^ = 0.95 ± 0.07 s^−1^ with TKI pretreatment (*p* < 0.05).

An additional parameter we measured was the fraction of colocalized molecules. Fig. 3 *d* shows the distribution of the percentage of detected dimers in the experiments described above. The fraction of gefitinib-pretreated dimers displayed a reduced variance of 1.95% ± 0.63% versus 3.59% ± 1.56% for the untreated sample, consistent with the reduction in *k*_off_.

Simulations were performed with parameters set to the experimentally determined values for intensity, background, photobleaching rate, and diffusion coefficient. Colocalization analysis of thousands of simulated single molecules, similar to the number of molecules tracked in a typical experiment, resulted in only ∼0.2% colocalized molecules, which is significantly below the experimentally observed colocalization rates (Fig. 3 *f*). Only five molecules were found to coincidentally colocalize in time and space, compared with more than 300 in a real experiment.

### Effect of an inhibitory anti-EGFR scFv on the receptor homodimerization

A different class of EGFR inhibitors is represented by monoclonal antibodies that block ligand binding. To investigate whether this EGFR-inhibiting strategy has the same stabilizing effect on the EGFR homodimer (Fig. S3), we employed a dual-labeled 425 Snap (scFv) anti-EGFR developed in a previous study (48). The 425 Snap (scFv) can be used to detect receptor dimerization, but does not trigger downstream effector phosphorylation as probed by Western blotting (Fig. S3 b). Similarly, pretreatment with TKIs (gefitinib and lapatinib) was shown to inhibit receptor phosphorylation (Fig. S4). Dimerization of 425 Snap (scFv) was observed with one-color single-molecule detection (Fig. S3 d) and the off-rates of the nonliganded receptors were determined using the two-color assay described above. EGF and 425 Snap (scFv) differed significantly in the percentage of colocalized single-molecule tracks (Fig. S3 f). The largest percentage of dimers per cell (2.59% ± 0.70%) was detected for EGF-mediated activation. When a combination of EGF and scFv ligands was used, the percentage of homodimers decreased to 1.01% ± 0.34%, with the lowest fraction of dimers recorded for scFv ligand only (0.40% ± 0.34%). Experiments with all three ligands were performed on the same day, thus eliminating day-to-day variability. A consistent shift with the same trend was registered in repeated experiments.

We calculated the dissociation rates for the molecules that dimerized when scFv or EGF and scFv were used as ligands. From repeated experiments, we calculated the mean dissociation rates of *k*_off_^EGF&scFv^ = 1.42 ± 0.16 s^−1^ (2798 dimerization events) and *k*_off_^scFv^ = 1.02 ± 0.45 s^−1^ (1869 dimerization events), where the errors represent the mean ± SE. Statistical analysis did not show any significant difference compared with EGF. Our results suggest there is no significant difference between the dissociation constants of EGFR homodimers with 1) double-ligand EGF occupancy, 2) asymmetric dimers of one ligand-bound monomer and one 425 scFv bound receptor, and 3) double 425 scFV ligated dimers.

We concluded that gefitinib pretreatment yielded a more stable EGF-bound EGFR dimer, whereas the scFv inhibitor did not exert any measurable effect. Both classes of inhibitors (TKIs and 425 scFv) resulted in a lower density of dimers on the cell surface and reduced heterogeneity (Figs. 2 *f* and S2 f). We note that the reduced probability of observing dimers when using 425 scFv (EGFR) as a ligand (Fig. S2 f) significantly limited our ability to determine the various off-rates. Additionally, 425 scFv may induce extracellular conformational changes that through the coupling of extra- and intracellular domains may affect EGFR homodimerization.

### Tyrosine phosphatase overexpression stabilizes the EGFR dimer

An alternative negative-feedback mechanism for regulating downstream signaling of EGFR is receptor dephosphorylation (68,69). We previously showed that the EGFR-interacting protein phosphatase DEP-1 decreases EGFR phosphorylation and inhibits EGFR internalization and downstream signaling (45). We determined the off-rates of EGF-stimulated dimers in inactive versus overexpressed GFP phosphatase DEP-1 in HCC1954 cells via single-molecule imaging to further characterize the role of phosphorylation in EGFR stability.

**Figure 4:**
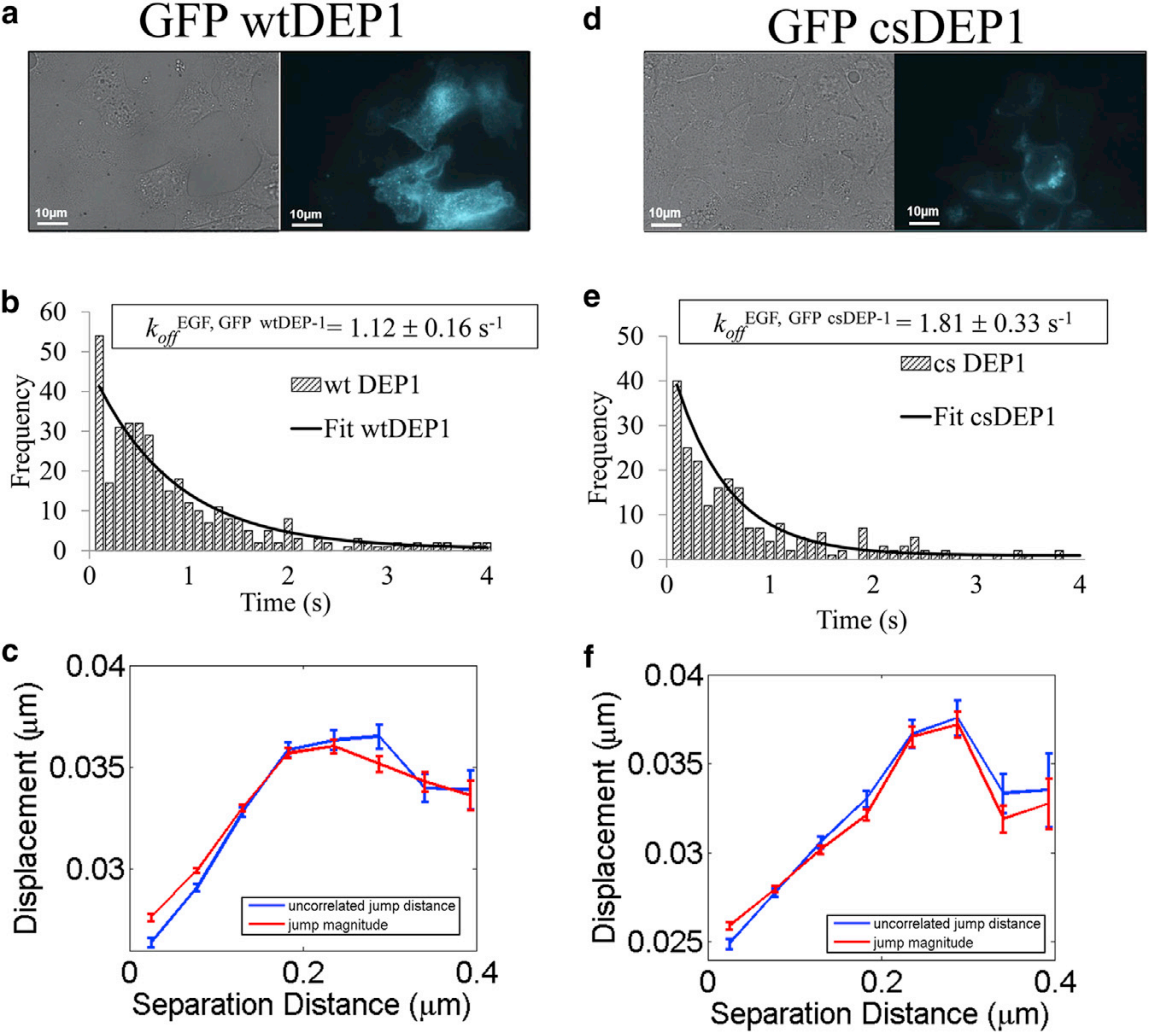
Overexpression of EGFR-targeted GFP DEP-1 phosphatase stabilized the EGFR homodimer. (*a*) Bright-field *trans* illumination (*left*) and epifluorescence (*right*) images show live cells and GFP fluorescence of the corresponding GFP WT DEP-1-transfected cell under 470 nm excitation. (*b*) Histograms of the durations of dimerization events for GFP WT DEP-1 in live HCC1954 cells. A monoexponential fit of the histogram yielded the off-rates *k*_off_^WTDEP-1^ = 1.12 ± 0.16 s^−1^ (382 events). (*c*) Ensemble correlated motion analysis for two-color EGF-treated HCC1954 cells overexpressing GFP WT DEP-1 phosphatase. Correlated motion at short distance can be visualized through the decrease in uncorrelated jump distance (*blue*). The concurrent drop in jump magnitude (*red*) indicates decreased diffusion for the homodimer. (*d–f*) Corresponding data for the GFP cs DEP-1 inactive phosphatase control experiment. The dimer off-rate was *k*_off_^csDEP-1^ = 1.81 ± 0.33 s^−1^ (248 events). To see this figure in color, go online.

Cells expressing the GFP DEP-1 construct were identified via GFP fluorescence excited with 470 nm light. Fig. 4, *a* and *d*, respectively, show transmitted bright-field illumination and 470 nm excited epifluorescence images of live cells transfected with WT DEP-1 and inactive cs DEP-1. Bright-field images show all of the cells present in the field of view. Only the GFP-transfected cells can be visualized in the corresponding epifluorescence image. GFP detection enabled us to select only the phosphatase DEP-1-transfected cells for data acquisition. The dissociation constants were derived as described above and the results are summarized in Table S2. Distribution of the homodimer lifetimes in cells expressing the WT and cs DEP-1 (EGFR) plasmids were fitted to a monoexponential as shown in Fig. 4, *b* and *e*, respectively. In the presence of cs DEP-1, the EGFR off-rate increased from *k*_off_^EGF,WTDEP-1^ = 1.12 ± 0.16 s^−1^ to *k*_off_^EGF,csDEP-1^ **=** 1.81 ± 0.33 s^−1^. Thus, EGFR dephosphorylation also has a stabilizing effect on the EGFR homodimer, similar to that observed for TKIs. Ensemble correlated motion analysis again demonstrated a decrease in uncorrelated motion and diffusion at small separations (Fig. 4, *c* and *f*).

### The cellular proliferative response to gefitinib depends on its ability to dimerize

**Figure 5:**
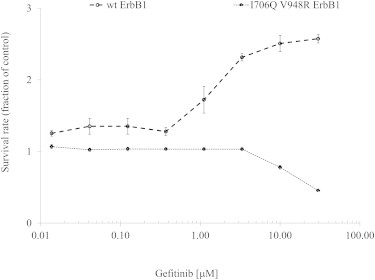
HCC1954 cells were treated for 96 h with a range of gefitinib concentrations and proliferation was measured using an Alamar Blue assay (Life Sciences). Cells were transfected with WT and I706Q/V948R ErbB1 24 h before treatment. Plotted are the means ± SE of a triplicate experiment. Data were normalized to control (untreated cells).

We postulated that the TKI-associated change in the EGFR homodimer kinetics could directly impact the signaling property of the EGFR signaling network on the plasma membrane and hence the proliferative activity of the breast tumor cells. The current models for intracellular EGFR homodimerization suggest the formation of an asymmetric dimer between the C-terminal lobe of one kinase domain (which acts as the activator) and the N-terminal lobe of another kinase domain (which acts as the receiver) (13,70,71). To test our hypothesis that the gefitinib-induced EGFR dimer may affect cell proliferative function, we studied the effect of gefitinib on the proliferation of HCC1954 cells expressing the WT and (I706Q, V948R) ErbB1 plasmids. In a recent study, we showed that the EGFR I706Q, V948R mutation perturbs the ability of EGFR to dimerize to another ErbB partner, ErbB4 (47). HCC1954 (which coexpresses both EGFR and HER2) was previously shown to be prone to developing resistance to EGFR/HER2 inhibition (72). The dose-response proliferation curve after 96 h of gefitinib treatment is shown in Fig. 5. Above 300 nM, gefitinib was shown to increase the proliferation of HCC1954 transfected with WT EGFR. Sensitivity to gefitinib (i.e., suppression of proliferation) was restored by expressing the dimerization-incompetent EGFR mutant.

## Discussion

Although recent studies have provided extensive information about the allosteric conformational change of EGFR that is induced by growth factor stimulation (3–7), and the use of single-molecule binding kinetics to monitor conformational changes in proteins is fairly well established (8), the effects of clinically used, targeted therapies against EGFR are only beginning to be understood (9,73). Furthermore, the advent of next-generation sequencing (NGS) (74) has revealed genetic mutations that are widely heterogeneous among tumor subclones, even within the same patient tumor (75,76). The use of quantitative, single-molecule-based imaging techniques to probe the effects of drug treatment on EGFR will complement the NGS approach by providing a functional readout that can potentially detect genetic variants (of different allelic fractions) that break through as the tumor subclones evolve.

The TKI gefitinib inhibits growth in some tumor types by targeting EGFR. Recurrent somatic activating mutations that occur in the exons encoding the kinase domain of EGFR have been reported to respond clinically to gefitinib (77–79). However, most patients who respond to therapy ultimately develop disease progression within 9–14 months of treatment. This particular aspect of receptor biology is very much in need of further elucidation within the ErbB therapeutic field, to improve our understanding of the heterogeneity in treatment response and ultimately to improve the efficacy of treatment for cancer patients.

Here, we have shown the effect of clinically used EGFR-targeted inhibitors (gefitinib and lapatinib) on the EGFR homodimerization kinetics in a basal-like breast cancer cell line, HCC1954. Gefitinib was shown to modulate EGFR homodimerization at the single-molecule level via a 25% increase in the duration of a homodimer in the presence of the drug. The stability conferred by gefitinib binding allows EGFR to form a higher fraction of homodimers. FRET/FLIM measurements showed a 40% increase in the fraction of homodimers in gefitinib-treated cells compared with nontreated cells. The ability of gefitinib to modulate EGFR homodimerization is likely to be important for cellular signaling. HCC1954 (which coexpresses both EGFR and HER2) was previously shown to be prone to developing resistance to EGFR/HER2 inhibition (72). The addition of gefitinib to WT EGFR at a concentration above 300 nM was shown to enhance cell proliferation, contrary to the intended role of TKIs. Sensitivity to gefitinib was restored when we used a double-site, dimerization-deficient EGFR mutant (the aforementioned EGFR I706Q, V948R mutation, which perturbs the ability of EGFR to dimerize through either the C-lobe (activator) or N-lobe (receiver) (47)). This suggests that the modest drug-induced increase in stability of the EGFR homodimer may have a significant biological impact on the tumor cell’s proliferation potential. Small ATP-competitive inhibitors specifically inhibit kinase activity by binding to the ATP-binding sites situated in the cytoplasmic domain of EGFR. However, the effect on the dissociation constant, percentage of homodimers, and cellular proliferation is not universal for all inhibitors and must be interpreted in the context of drug-bound receptor conformation. In the case of type I inhibitors such as gefitinib, the crystal structure (Protein Data Bank ID 2ITY) shows that the drug stabilizes the active EGFR conformation (as defined by the model of an active, asymmetric, head-to-tail EGFR homodimer (80)). Upon gefitinib pretreatment, catalytically incompetent quasi-dimers are formed (34). Lapatinib, which is a type II inhibitor, stabilizes the inactive EGFR conformation. We found that the observed effect of lapatinib on the homodimer stoichiometry and dissociation constant was not statistically significant (data not shown). This is similar to another EGFR TKI, PD153035, which was previously reported to have no effect on the ligand-bound EGFR homodimer off-rates (24). However, no crystallographic structure of the PD153035-bound receptor is currently available for comparison with that of gefitinib/lapatinib.

The overall architecture of the ErbB pathway, which has been described as a bow-tie (or hourglass)-shaped structure with several feedback loops (81,82), is most likely to be the basis for the amplification of the modest drug-induced increase in stability of the EGFR homodimer. Also, previous single-molecule studies have shown that dynamic lateral interactions among EGFR receptors on the plasma membrane induce amplification of EGFR signaling (83). This is consistent with our observation that a small change in the duration of the homodimer with gefitinib treatment resulted in a significant increase in the fraction of asymmetric, unphosphorylated receptor homodimers. This is in addition to the link we have shown between the ability of EGFR to undergo dimerization and the impact of gefitinib on tumor cell proliferation. Similarly, a small change in the duration of an EGFR-containing heterodimer can result in a large downstream effect. As an example, a similar amplification effect was recently observed for the EGFR-ErbB4 heterodimer (47). The effect of dimer-perturbing mutations (including the I706Q, V948R double mutation of EGFR used in this study) on the heterodimer off-rate was modest (∼33% increase), but resulted in the abrogation of growth factor-stimulated cell migration.

The inhibitory 425 SNAP (scFv), which can block endogenous EGF binding, caused a reduction in the percentage of EGFR single-molecule tracks that colocalized. For the dimers that did manage to form, however, the off-rate was not significantly different from that observed with the natural ligand EGF. This is different from the unliganded EGFR homodimer, which was found to have a >4-fold faster *k*_off_, as detected by the camelid variable fragment of heavy chain antibody, which does not compete for EGF binding (24). The difference between the different antibodies may be accounted for by the different receptor conformations these antibodies can stabilize (according to the effect on EGF binding), similar to the case of the different TKIs described above. Up to 90% of the dimers formed when scFV only was used as ligand were inactive and would be most likely be represented by a symmetric TKD. The similar dissociation rates measured for EGF- and scFv-induced dimers suggest that both symmetric and asymmetric TK interfaces are equally able to stabilize the intracellular part of the dimer.

In the case of overexpressed DEP-1 tyrosine phosphatase, the levels of EGFR phosphorylation are determined by the balance between the receptor TK and phosphatase activities. The increase in apparent FRET efficiency in cells overexpressing phosphatase could only be observed in the absence of EGF stimulation. The increased homodimer stability with phosphatase overexpression could be observed in single-molecule experiments performed in the presence of picomolar concentrations of the ligand, an order of magnitude below the concentration used in ensemble measurements. Cells overexpressing EGFR-specific TK DEP-1 phosphatase showed increased dimer stability (*k*_off_^WTDEP-1^ = 1.12 ± 0.16 s^−1^) as compared with EGFR receptor homodimers in cells transfected with the inactive DEP-1 phosphatase (*k*_off_^csDEP-1^ = 1.81 ± 0.33 s^−1^). The measured difference between the dissociation rate of the phosphorylated and dephosphorylated EGFR homodimers may be attributed to different conformations of the phosphorylated versus nonphosphorylated receptor.

We also show some examples of spFRET recorded on EGFR homodimers stained with donor and acceptor cytoplasmic antibody D38-B1 with and without TKI pretreatment (Fig. 1, *d* and *e*). spFRET was visualized via donor recovery after acceptor photobleaching in fixed HCC1954 cells. Detection of spFRET demonstrates direct interaction of the individual monomers, providing further support for the conclusion that our single-molecule results from live cells (with or without TKI) report on bona fide complexes rather than two molecules coconfined within a diffraction-limited spot. However, quantification of the spFRET is beyond of the scope of this work.

Previous studies (84,85) showed that when EGFR phosphorylation was prevented by cytoplasmic mutations, the dimer’s binding affinity for ligand was higher than that predicted by a ligand-induced dimerization model. Our single-molecule data extend that observation by demonstrating directly that the stability of the EGFR homodimer is sensitive to the phosphorylation state of the receptor. A recent study also noted the correlation between suppressed EGFR autophosphorylation on specific binding sites (Grb2 and c-Cbl) and receptor stability and sustained signaling (47,86). Taken together, our observations (obtained using TKIs and phosphatase expression) provide support for the idea proposed in other reports that there are conformational differences between the phosphorylated and unphosphorylated forms of the receptor.

## Supporting Material

Document S1. Supporting Materials and Methods, Supporting Results, four figures, and two tables

Document S2. Article plus Supporting Material

## Supporting Citations

Reference (87) appears in the Supporting Material.

## References

[1] Schlessinger J. (2002). Ligand-induced, receptor-mediated dimerization and activation of EGF receptor. Cell.

[2] Carpenter G., Stoscheck C.M., Soderquist A.M. (1982). Epidermal growth factor. Ann. N. Y. Acad. Sci..

[3] Kumar A., Petri E.T., Boggon T.J. (2008). Structure and clinical relevance of the epidermal growth factor receptor in human cancer. *J. Clin.* On*col.*.

[4] Ross J.S., Fletcher J.A. (1998). The HER-2/neu oncogene in breast cancer: prognostic factor, predictive factor, and target for therapy. Stem Cells.

[5] Selvaggi G., Novello S., Scagliotti G.V. (2004). Epidermal growth factor receptor overexpression correlates with a poor prognosis in completely resected non-small-cell lung cancer. Ann. Oncol..

[6] Ogiso H., Ishitani R., Yokoyama S. (2002). Crystal structure of the complex of human epidermal growth factor and receptor extracellular domains. Cell.

[7] Lemmon M.A., Schlessinger J. (2010). Cell signaling by receptor tyrosine kinases. Cell.

[8] Ferguson K.M. (2004). Active and inactive conformations of the epidermal growth factor receptor. Biochem. Soc. Trans..

[9] Moriki T., Maruyama H., Maruyama I.N. (2001). Activation of preformed EGF receptor dimers by ligand-induced rotation of the transmembrane domain. J. Mol. Biol..

[10] Clayton A.H., Walker F., Burgess A.W. (2005). Ligand-induced dimer-tetramer transition during the activation of the cell surface epidermal growth factor receptor-A multidimensional microscopy analysis. J. Biol. Chem..

[11] Lemmon M.A., Bu Z., Schlessinger J. (1997). Two EGF molecules contribute additively to stabilization of the EGFR dimer. EMBO J..

[12] Jura N., Endres N.F., Kuriyan J. (2009). Mechanism for activation of the EGF receptor catalytic domain by the juxtamembrane segment. Cell.

[13] Zhang X., Gureasko J., Kuriyan J. (2006). An allosteric mechanism for activation of the kinase domain of epidermal growth factor receptor. Cell.

[14] Alvarado D., Klein D.E., Lemmon M.A. (2010). Structural basis for negative cooperativity in growth factor binding to an EGF receptor. Cell.

[15] Garrett T.P., McKern N.M., Ward C.W. (2003). The crystal structure of a truncated ErbB2 ectodomain reveals an active conformation, poised to interact with other ErbB receptors. Mol. Cell.

[16] Garrett T.P., McKern N.M., Ward C.W. (2002). Crystal structure of a truncated epidermal growth factor receptor extracellular domain bound to transforming growth factor *α*. Cell.

[17] Ferguson K.M. (2008). Structure-based view of epidermal growth factor receptor regulation. Annu. Rev. Biophys..

[18] Stamos J., Sliwkowski M.X., Eigenbrot C. (2002). Structure of the epidermal growth factor receptor kinase domain alone and in complex with a 4-anilinoquinazoline inhibitor. J. Biol. Chem..

[19] Wood E.R., Truesdale A.T., Shewchuk L. (2004). A unique structure for epidermal growth factor receptor bound to GW572016 (Lapatinib): relationships among protein conformation, inhibitor off-rate, and receptor activity in tumor cells. Cancer Res..

[20] Yun C.H., Boggon T.J., Eck M.J. (2007). Structures of lung cancer-derived EGFR mutants and inhibitor complexes: mechanism of activation and insights into differential inhibitor sensitivity. Cancer Cell.

[21] Red Brewer M., Choi S.H., Carpenter G. (2009). The juxtamembrane region of the EGF receptor functions as an activation domain. Mol. Cell.

[22] Burgess A.W., Cho H.S., Yokoyama S. (2003). An open-and-shut case? Recent insights into the activation of EGF/ErbB receptors. Mol. Cell.

[23] Dixit A., Verkhivker G.M. (2009). Hierarchical modeling of activation mechanisms in the ABL and EGFR kinase domains: thermodynamic and mechanistic catalysts of kinase activation by cancer mutations. PLOS Comput. Biol..

[24] Low-Nam S.T., Lidke K.A., Lidke D.S. (2011). ErbB1 dimerization is promoted by domain co-confinement and stabilized by ligand binding. Nat. Struct. Mol. Biol..

[25] Hiroshima M., Saeki Y., Sako Y. (2012). Dynamically varying interactions between heregulin and ErbB proteins detected by single-molecule analysis in living cells. Proc. Natl. Acad. Sci. USA.

[26] Teramura Y., Ichinose J., Sako Y. (2006). Single-molecule analysis of epidermal growth factor binding on the surface of living cells. EMBO J..

[27] Webb S.E., Roberts S.K., Martin-Fernandez M.L. (2008). Single-molecule imaging and fluorescence lifetime imaging microscopy show different structures for high- and low-affinity epidermal growth factor receptors in A431 cells. Biophys. J..

[28] Yu C., Hale J., Irudayaraj J. (2009). Receptor overexpression or inhibition alters cell surface dynamics of EGF-EGFR interaction: new insights from real-time single molecule analysis. Biochem. Biophys. Res. Commun..

[29] Kawashima N., Nakayama K., Biju V. (2010). Reversible dimerization of EGFR revealed by single-molecule fluorescence imaging using quantum dots. Chemistry.

[30] Sako Y., Minoghchi S., Yanagida T. (2000). Single-molecule imaging of EGFR signalling on the surface of living cells. Nat. Cell Biol..

[31] Xiao Z., Zhang W., Fang X. (2008). Single-molecule diffusion study of activated EGFR implicates its endocytic pathway. Biochem. Biophys. Res. Commun..

[32] Chen J., Irudayaraj J. (2010). Fluorescence lifetime cross correlation spectroscopy resolves EGFR and antagonist interaction in live cells. Anal. Chem..

[33] Vivanco I., Robins H.I., Mellinghoff I.K. (2012). Differential sensitivity of glioma- versus lung cancer-specific EGFR mutations to EGFR kinase inhibitors. Cancer Discov..

[34] Bublil E.M., Pines G., Yarden Y. (2010). Kinase-mediated quasi-dimers of EGFR. FASEB J..

[35] Lu C., Mi L.-Z., Springer T.A. (2012). Mechanisms for kinase-mediated dimerization of the epidermal growth factor receptor. J. Biol. Chem..

[36] Shankaran H., Wiley H.S., Resat H. (2006). Modeling the effects of HER/ErbB1-3 coexpression on receptor dimerization and biological response. Biophys. J..

[37] Shankaran H., Zhang Y., Resat H. (2008). Quantifying the effects of co-expressing EGFR and HER2 on HER activation and trafficking. Biochem. Biophys. Res. Commun..

[38] Mustafa M., Mirza A., Kannan N. (2011). Conformational regulation of the EGFR kinase core by the juxtamembrane and C-terminal tail: a molecular dynamics study. Proteins.

[39] Park J.H., Liu Y., Radhakrishnan R. (2012). Erlotinib binds both inactive and active conformations of the EGFR tyrosine kinase domain. Biochem. J..

[40] Harms B.D., Kearns J.D., Schoeberl B. (2012). Optimizing properties of antireceptor antibodies using kinetic computational models and experiments. Methods Enzymol..

[41] Schoeberl B., Eichler-Jonsson C., Müller G. (2002). Computational modeling of the dynamics of the MAP kinase cascade activated by surface and internalized EGF receptors. Nat. Biotechnol..

[42] Arkhipov A., Shan Y., Shaw D.E. (2013). Architecture and membrane interactions of the EGF receptor. Cell.

[43] Nielsen U.B., Schoeberl B. (2005). Using computational modeling to drive the development of targeted therapeutics. IDrugs..

[44] Pryor M.M., Low-Nam S.T., Edwards J.S. (2013). Dynamic transition states of ErbB1 phosphorylation predicted by spatial stochastic modeling. Biophys. J..

[45] Tarcic G., Boguslavsky S.K., Yarden Y. (2009). An unbiased screen identifies DEP-1 tumor suppressor as a phosphatase controlling EGFR endocytosis. Curr. Biol..

[46] Gazdar A.F., Kurvari V., Shay J.W. (1998). Characterization of paired tumor and non-tumor cell lines established from patients with breast cancer. Int. J. Cancer..

[47] Kiuchi T., Ortiz-Zapater E., Ng T. (2014). The ErbB4 CYT2 variant protects EGFR from ligand-induced degradation to enhance cancer cell motility. Sci. Signal..

[48] Kampmeier F., Niesen J., Thepen T. (2010). Rapid optical imaging of EGF receptor expression with a single-chain antibody SNAP-tag fusion protein. Eur. J. Nucl. Med. Mol. Imaging.

[49] Barber P.R., Tullis I.D., Vojnovic B. (2013). The Gray Institute ‘open’ high-content, fluorescence lifetime microscopes. J. Microsc..

[50] Barber P.R., Ameer-Beg S.M., Vojnovic B. (2009). Multiphoton time-domain fluorescence lifetime imaging microscopy: practical application to protein-protein interactions using global analysis. J. R. Soc. Interface.

[51] Ng T., Squire A., Parker P.J. (1999). Imaging protein kinase C*α* activation in cells. Science.

[52] Morris J.R., Boutell C., Solomon E. (2009). The SUMO modification pathway is involved in the BRCA1 response to genotoxic stress. Nature.

[53] Carlin L.M., Evans R., Ng T. (2011). A targeted siRNA screen identifies regulators of Cdc42 activity at the natural killer cell immunological synapse. Sci. Signal..

[54] Fruhwirth G.O., Fernandes L.P., Ng T. (2011). How Förster resonance energy transfer imaging improves the understanding of protein interaction networks in cancer biology. ChemPhysChem.

[55] Lu H.P., Xun L., Xie X.S. (1998). Single-molecule enzymatic dynamics. Science.

[56] Press W.H., Teukolsky S.A., Flannery B.P. (1992). Numerical Recipes in C.

[57] Andrews N.L., Lidke K.A., Lidke D.S. (2008). Actin restricts FcepsilonRI diffusion and facilitates antigen-induced receptor immobilization. Nat. Cell Biol..

[58] Steinkamp M.P., Low-Nam S.T., Wilson B.S. (2014). erbB3 is an active tyrosine kinase capable of homo- and heterointeractions. Mol. Cell. Biol..

[59] Rolfe D.J., McLachlan C.I., Hobson M.P. (2011). Automated multidimensional single molecule fluorescence microscopy feature detection and tracking. Eur. Biophys. J..

[60] Barber P.R., Ameer-Beg S.M., Vojnovic B. (2005). Global and pixel kinetic data analysis for FRET detection by multi-photon time-domain FLIM. Proc. SPIE..

[61] Shu D., Zhang H., Guo P. (2007). Counting of six pRNAs of phi29 DNA-packaging motor with customized single-molecule dual-view system. EMBO J..

[62] Coban O., Lamb D.C., Nienhaus G.U. (2006). Conformational heterogeneity in RNA polymerase observed by single-pair FRET microscopy. Biophys. J..

[63] Defize L.H., Arndt-Jovin D.J., de Laat S.W. (1988). A431 cell variants lacking the blood group A antigen display increased high affinity epidermal growth factor-receptor number, protein-tyrosine kinase activity, and receptor turnover. J. Cell Biol..

[64] Defize L.H., Boonstra J., de Laat S.W. (1989). Signal transduction by epidermal growth factor occurs through the subclass of high affinity receptors. J. Cell Biol..

[65] Suzuki K.G., Fujiwara T.K., Kusumi A. (2007). Dynamic recruitment of phospholipase C gamma at transiently immobilized GPI-anchored receptor clusters induces IP3-Ca^2+^ signaling: single-molecule tracking study 2. J. Cell Biol..

[66] Zanetti-Domingues L.C., Tynan C.J., Martin-Fernandez M. (2013). Hydrophobic fluorescent probes introduce artifacts into single molecule tracking experiments due to non-specific binding. *PLoS* ON*E*.

[67] Suzuki K.G., Kasai R.S., Kusumi A. (2012). Transient GPI-anchored protein homodimers are units for raft organization and function. Nat. Chem. Biol..

[68] Offterdinger M., Georget V., Bastiaens P.I. (2004). Imaging phosphorylation dynamics of the epidermal growth factor receptor. J. Biol. Chem..

[69] Reynolds A.R., Tischer C., Bastiaens P.I. (2003). EGFR activation coupled to inhibition of tyrosine phosphatases causes lateral signal propagation. Nat. Cell Biol..

[70] Zhang X., Pickin K.A., Kuriyan J. (2007). Inhibition of the EGF receptor by binding of MIG6 to an activating kinase domain interface. Nature.

[71] Zhang H., Berezov A., Greene M.I. (2007). ErbB receptors: from oncogenes to targeted cancer therapies. J. Clin. Invest..

[72] Wilson T.R., Fridlyand J., Settleman J. (2012). Widespread potential for growth-factor-driven resistance to anticancer kinase inhibitors. Nature.

[73] Tsai C.J., Nussinov R. (2013). The molecular basis of targeting protein kinases in cancer therapeutics. Semin. Cancer Biol..

[74] Stead L.F., Sutton K.M., Rabbitts P. (2013). Accurately identifying low-allelic fraction variants in single samples with next-generation sequencing: applications in tumor subclone resolution. Hum. Mutat..

[75] Burrell R.A., McGranahan N., Swanton C. (2013). The causes and consequences of genetic heterogeneity in cancer evolution. Nature.

[76] Gerlinger M., Rowan A.J., Swanton C. (2012). Intratumor heterogeneity and branched evolution revealed by multiregion sequencing. N. Engl. J. Med..

[77] Lynch T.J., Bell D.W., Haber D.A. (2004). Activating mutations in the epidermal growth factor receptor underlying responsiveness of non-small-cell lung cancer to gefitinib. N. Engl. J. Med..

[78] Paez J.G., Jänne P.A., Meyerson M. (2004). EGFR mutations in lung cancer: correlation with clinical response to gefitinib therapy. Science.

[79] Pao W., Chmielecki J. (2010). Rational, biologically based treatment of EGFR-mutant non-small-cell lung cancer. Nat. Rev. Cancer.

[80] Jura N., Shan Y., Kuriyan J. (2009). Structural analysis of the catalytically inactive kinase domain of the human EGF receptor 3. Proc. Natl. Acad. Sci. USA.

[81] Citri A., Yarden Y. (2006). EGF-ERBB signalling: towards the systems level. Nat. Rev..

[82] Oda K., Matsuoka Y., Funahashi A., Kitano H. (2005). A comprehensive pathway map of epidermal growth factor receptor signaling. Mol. Syst. Biol..

[83] Ichinose J., Murata M., Sako Y. (2004). EGF signalling amplification induced by dynamic clustering of EGFR. Biochem. Biophys. Res. Commun..

[84] Macdonald J.L., Pike L.J. (2008). Heterogeneity in EGF-binding affinities arises from negative cooperativity in an aggregating system. Proc. Natl. Acad. Sci. USA.

[85] Macdonald-Obermann J.L., Pike L.J. (2009). The intracellular juxtamembrane domain of the epidermal growth factor (EGF) receptor is responsible for the allosteric regulation of EGF binding. J. Biol. Chem..

[86] Hartman Z., Zhao H., Agazie Y.M. (2013). HER2 stabilizes EGFR and itself by altering autophosphorylation patterns in a manner that overcomes regulatory mechanisms and promotes proliferative and transformation signaling. On*cogene*.

[87] Hickinson D.M., Klinowska T., Ogilvie D. (2010). AZD8931, an equipotent, reversible inhibitor of signaling by epidermal growth factor receptor, ERBB2 (HER2), and ERBB3: a unique agent for simultaneous ERBB receptor blockade in cancer. Clin. Cancer Res..

