# Supplementary material for: Effect of Phosphorylation on EGFR Dimer Stability Probed by Single-Molecule Dynamics and FRET/FLIM
Source: Biophys J. 2015 Mar 10;108(5):1013–26. doi: 10.1016/j.bpj.2015.01.005 (PMC4375452; doi:10.1016/j.bpj.2015.01.005)
Supplement: Document S1. Supporting Materials and Methods, Supporting Results, four figures, and two tables [file mmc1.pdf]

SUPPORTING MATERIALS AND METHODS

Western blotting of EGFR phosphorylation

Hcc1954 cells were seeded onto a six-well plate and cultured as described in the main text. At about 70 % confluence, cells were serum-starved for 2h at 37°C under 5 % CO_2. TKI treated cells were incubated with 10 µM AZD8931 (Stratech Scientific Ltd., U.K.) for 1h at 37°C. Then, samples were treated with 1.6 nM EGF or 18 nM 425 Snap scFv in media without fetal bovine serum for 30 min at 37°C. Cell lysates were prepared by adding 100 µL of lysis buffer (200 mM Tris, 2.5 % SDS, 50 % glycerol) to each well and collecting the scraped cells. The cell lysates were heated up for 15 min at 95°C and allowed to gradually cool down to room temperature. Cellular proteins were separated on a 10 % SDS-polyacrylamide gel and then transferred onto a nitrocellulose membrane. Further, the membrane was blocked with 4 % BSA in TBS for 1h and incubated overnight at 4°C with primary antibody diluted in 0.5 % BSA in TBS-T (0.02 M Tris-HCl, pH 7.8, 0.15M NaCl, 0.05 % Tween 20). EGFR downstream signaling was probed via ERK. pERK1/2 was obtained from New England Biolabs, UK, and total ERK was purchased from Sigma, UK. Immunoreactive bands were detected by standard ECL reaction.

Competitive binding analysis by flow cytometry

A431 cells were incubated for 30 min on ice with various amounts of 425 Snap (scFv) (0.83 - 830 nM) prior to the addition of 0.125 nM Snap EGF-BG-505 competitor. The fluorescence intensity of the cells was measured by flow cytometry (FACS calibur, Backton Dickinson, UK) and the median, relative value plotted against the concentration of competitor. Data were normalized with the untreated cells as 0 % binding while the labelled probe without competitor was 100 % binding. A sigmoidal dose response curve fit was used to calculate EC_{50} values with GraphPad software (Prism).

Confocal fluorescence imaging

Confocal images were obtained from an upright Zeiss LSM 510 META confocal microscope (Carl Zeiss, UK). Excitation of Alexa 546 Fluor was achieved using the 543 nm line of the single photon HeNe laser. Nuclear fluorescent staining with Hoechst was visualized with the 488 nm spectral line of an Ar laser. A plan-apochromat oil objective 63×, NA = 1.4 and the appropriate filters were used for the collection of images. Image processing was performed using the LSM 510 software.

SUPPORTING RESULTS

Colocalization durations were corrected for the fluorophores photobleaching according to the equation $\tau = (\tau_{app}^{-1} - \tau_1^{-1} - \tau_2^{-1})^{-1}$, where τ_{app} is the apparent colocalization time, and τ_1 and τ_2 are the times to photobleaching for the individual fluorescent probes used in the two-color single-molecule experiments.

EGFR homodimer	Fluorescent ligands	Photobleaching lifetime (s)	Apparent colocalization duration (s)	Photobleaching corrected colocalization duration (s)
Hcc1954	Alexa 546 EGF	28.76	0.85	0.90
	Atto 647 EGF	39.27		
Gefitinib pre-treated Hcc1954	Alexa 546 EGF	34.38	1.05	1.14
	Atto 647 EGF	22.80		
GFP csDEP-1 transfected Hcc1954	Alexa 546 EGF	27.84	0.53	0.55
	Atto 647 EGF	26.22		
GFP wtDEP-1 Transfected Hcc 1954	Alexa 546 EGF	24.69	0.83	0.88
	Atto 647 EGF	28.82		

Table S1. Single-molecule experimentally determined and photo bleaching corrected colocalization durations.

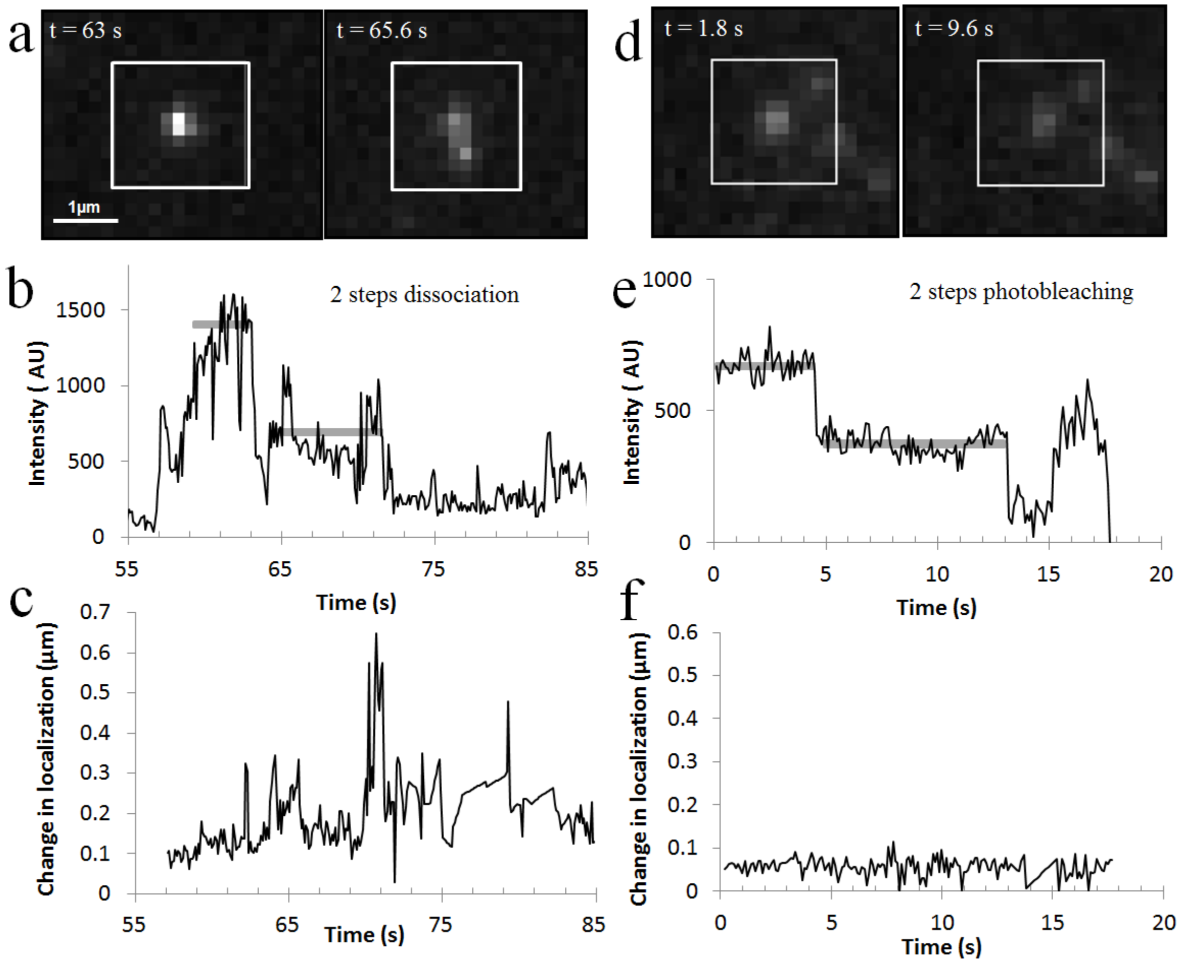

Figure S1. Direct visualisation of EGFR dimerization *in situ* detected by one-color single-molecule tracking. **a.** Snapshot of a single receptor homodimer (*left*) and its dissociation into the individual monomers (*right*); **b.** Fluorescence intensity trace of the tracked dimer shown in **a.** The fluorescence intensity at t = 63 s when the dimer image was captured is approximatelly double of the intensity of the individual monomer captured at t = 65.5 s from the beginning of the measurement. All images have the 1 μm same scale bar; **c.** Correlated motion plot for the trace depicted in Fig. S1b. **d.** Corresponding images of a photobleaching immobile molecule attached to the glass; **e.** Two-steps photobleaching trace of the molecule shown in **d.**; **f.** Position plot of a static molecule displayed on the same scale as for the dynamic dimer shows the level of accuracy in determining the location of individual molecules.

One-color visualization in real time of association/dissociation events

To visually illustrate the capability of our single-molecule tracking method to record *in situ* EGFR dimerization we present two snapshots of a single channel detected feature at time t = 63 s from the beginning of the measurement (Fig. S1a, *left*) and 2.6 s later (Fig. S1a, *right*). At t = 63 s, the snapshot depicts an EGFR dimer with double Atto 647 EGF ligand occupancy. At t = 65.6 s, the dimer has dissociated and the individual monomers can be seen as two separate dots with approximately half the intensity of the dimer. The white square represents the ROI considered around each pixel for the Bayesian segmentation. Fig. S1b shows the two distinct fluorescence intensity levels corresponding to a dimer and one of the monomers upon dissociation of its dimerization partner. In this example, the detected feature was an EGFR dimer which dissociated

into the individual monomers during the tracking. The original track followed only one of the monomers while the other dissociated monomer would have been resolved by a different track. The change in the detected feature location is plotted as a function of time in Fig. S1c to illustrate the distance covered by an EGFR dimer (the feature initially detected from the beginning of the measurement until time t = 65.6 s) and monomer (the feature detected after the dissociation of one of the monomers at t = 65.6 s) during our acquisition time. As one of the monomers dissociated it led to a brief but significant background variation in the ROI surrounding the detected feature and hence the calculated feature position which appeared as a spike in the feature position plot (Fig. S1c).

For comparison, we also present single-molecule images of immobilized molecules carrying two identical fluorophores as displayed in Fig. S1d before (*left*) and after (*right*) photobleaching of the 1st fluorophore. The corresponding two-step photobleaching trace is shown in Fig. S1e. The immobilized feature position was plotted as a function of time on the same scale as the homodimer position (Fig. S1f) to evaluate the displacement of functional receptors versus the experimental noise in localization precision. As expected, the position of the immobile feature labelled with two fluorophores remained constant throughout the measurement displaying only small fluctuations within the noise level.

Effect of an inhibitory anti-EGFR scFv on the receptor homodimerization

Cell membrane staining after incubation for 15 min at 37°C with Alexa Fluor 546 scFv or EGF&Alexa 546 Fluor scFv is shown in Fig. S3a. The biological activity of 425 Snap (scFv) was evaluated by probing EGFR downstream signaling via pERK1/2. Hcc1954 cells were incubated for 15 min at 37°C with 18 nM 425 Snap (scFv) or 1.6 nM EGF, with and without pre-treatment with AZD 8931 inhibitor, a HER1/2/3 TKI (2). The cell lysates were analyzed by western blotting with anti-phosphotyrosine 1173 phospho-ERK or anti-ERK as loading control (Fig. S3b). 425 Snap (scFv) had minimal effect on EGFR signaling as compared to its natural ligand, providing that the differences in the total ERK concentration were accounted for. Inhibition of basal levels of ERK1/2 phosphorylation was observed when either 425 Snap (scFv) or EGF stimulated cells were pre-treated with AZD 8931.

To examine the effect of 425 Snap (scFv) on EGF binding, we performed a binding competition assay by flow cytometry using BG-505 labelled Snap EGF. Cells were incubated for 30 min on ice with varying amounts of 425 Snap (scFv) before the addition of labelled Snap EGF. The percentage of Snap EGF binding cells was plotted against the competitor concentration and fitted to a sigmoidal curve with a half-maximum effective concentration of 82 nM indicating competitive binding (Fig. S3c).

We illustrate in Fig. S3d the assembly of a scFv-bound dimer captured by one-color single-molecule imaging. Binding of a second fluorescent molecule appears as a step increase in the fluorescence intensity of the molecule which was detected first. The corresponding trajectory of the detected feature is shown in Fig. S3e.

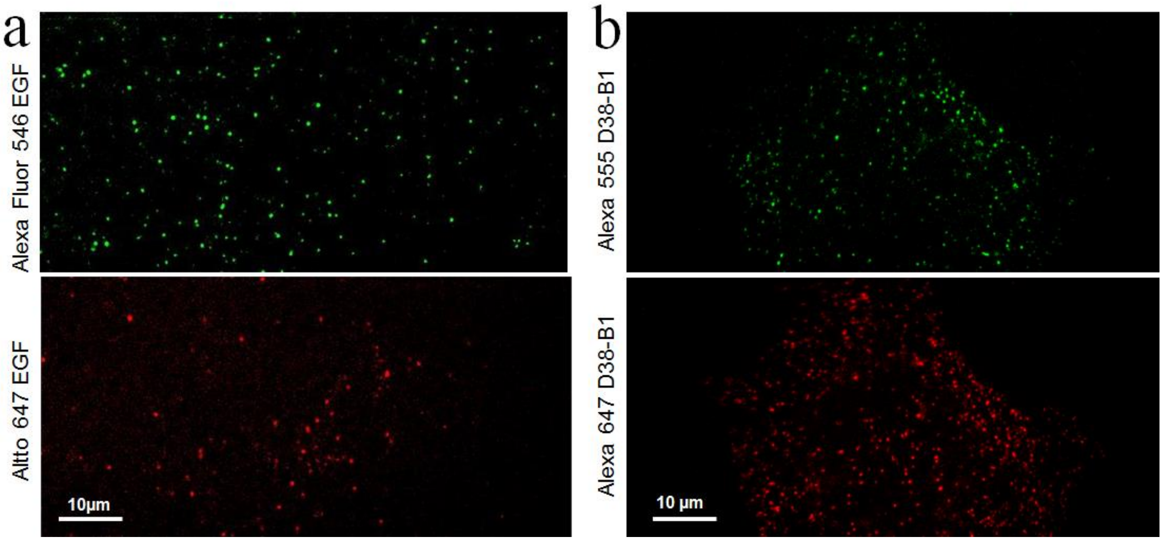

Figure S2. Single-molecule total internal reflection fluorescence images of **a.** intact, endogeneous EGFRs dual-labeled with Alexa Fluor 546 EGF (green) and Atto 647 EGF (red), respectively in live Hcc1954 breast cancer cells and **b.** EGF stimulated fixed cells immunostained with Alexa Fluor 555 (green) and Alexa Fluor 647 (red) D38-B1 anti-EGFR

TKIs	ErbB1 off rate (s^{-1})	
	EGF	EGF+TKI
Lapatinib	1.22	0.95
Lapatinib	1.20	0.94
Gefitinib	1.22	0.92
Gefitinib	1.57	1.09
AG1478	1.22	1.02
DEP-1 phosphatase overexpression	csDEP-1	wtDEP-1
	1.89	1.20
	1.64	1.31

Table S2. Off-rates of EGFR homodimer by condition demonstrates formation of more stable dimers with various TKIs pre-treatment and over-expression of wt DEP-1 as compared to no treatment or over-expression of inactive phosphatase cs DEP-1, respectively. All measurements were performed in the presence of Alexa 546 EGF and Atto 647 EGF.

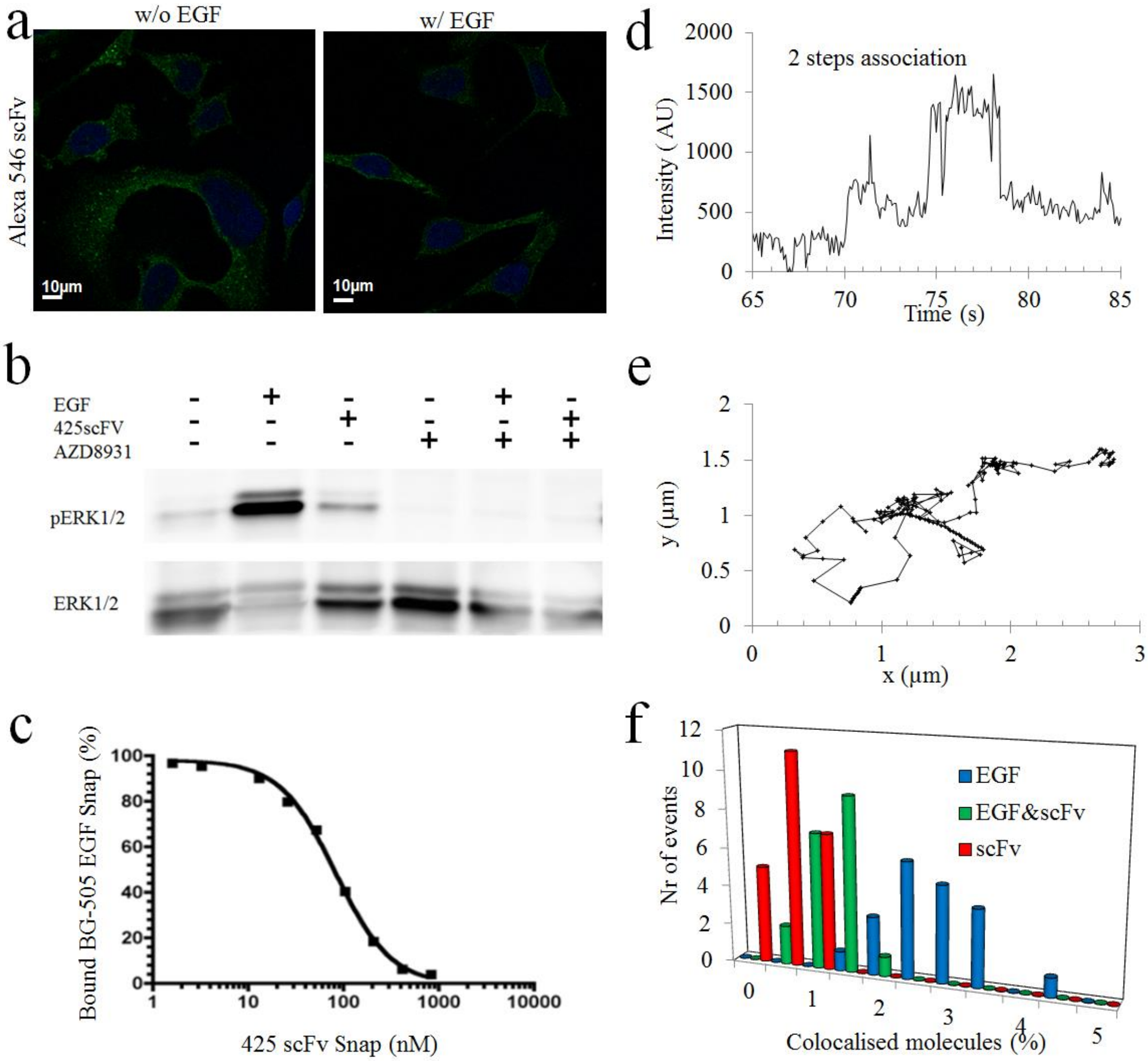

Figure S3. Effect of an inhibitory anti-EGFR 425 Snap (scFv) on receptor homodimerization. **a.** Fluorescence immunostaining of Hcc1954 cells upon 30 min incubation at 37°C with Alexa 546 425 Snap (scFv) (*left*) and EGF&Alexa 546 425 Snap (scFv) (*right*); **b.** Western blot analysis of downstream EGFR induced ERK phosphorylation when stimulated with EGF or 425 Snap (scFv) with or without pre-treatment with AZD 8931. The phosphorylation was probed and quantified via pERK. A 90 % reduction in phosporylation was detected when 425 Snap (scFv) was used as a ligand as compared to EGF alone. **c.** Competitive binding assay between 425 Snap (scFv) and Snap EGF. Cells were incubated for 30 min at 37°C with varying amounts of 425 Snap scFv (0.83 – 830 nM) before the addition of labelled EGF. Cell bound, fluorescently labelled Snap EGF was displaced by 425 Snap (scFv) with an IC_{50} of 82 nM, as determined by FACS; **d** and **e.** A fluorescence intensity trace recorded for the association of two scFv bound receptors and its corresponding trajectory to illustrate the range of motion. **f.** Percentage of EGFR homodimers depends on the nature of the ligand. The highest percentage of homodimers was observed for the activating EGF ligand (3.59 ± 1.56) followed by a mixture of the activating and non-activating ligands EGF&scFv (1.95 ± 0.63) and lastly the non-activating scFv (1.73 ± 0.49).

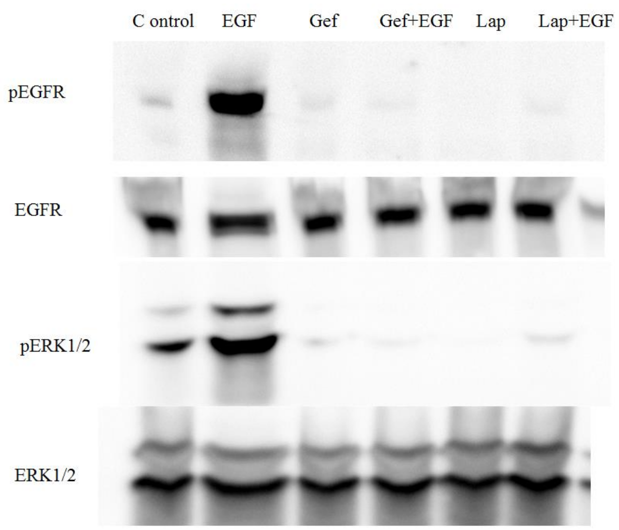

Figure S4. Effect of gefitinib and lapatinib treatment on EGFR phosphorylation and EGFR induced ERK phosphorylation, as detected by immunoblotting with phospho-specific antibodies. EGF stimulation was used as positive control. Inhibition of phosphorylation was observed with both gefitinib and lapatinib pre- treatment, with or without EGF stimulation.

SUPPORTING REFERENCES

1. Rolfe, D. J., C. I. McLachlan, M. Hirsch, S. R. Needham, C. J. Tynan, S. E. Webb, M. L. Martin-Fernandez, and M. P. Hobson. 2011. Automated multidimensional single molecule fluorescence microscopy feature detection and tracking. European biophysics journal : EBJ 40:1167-1186.
2. Hickinson, D. M., T. Klinowska, G. Speake, J. Vincent, C. Trigwell, J. Anderton, S. Beck, G. Marshall, S. Davenport, R. Callis, E. Mills, K. Grosios, P. Smith, B. Barlaam, R. W. Wilkinson, and D. Ogilvie. 2010. AZD8931, an equipotent, reversible inhibitor of signaling by epidermal growth factor receptor, ERBB2 (HER2), and ERBB3: a unique agent for simultaneous ERBB receptor blockade in cancer. Clinical cancer research : an official journal of the American Association for Cancer Research 16:1159-1169.
